# Resveratrol accumulation and its involvement in stilbene synthetic pathway of Chinese wild grapes during berry development using quantitative proteome analysis

**DOI:** 10.1038/s41598-017-10171-x

**Published:** 2017-08-24

**Authors:** Ruimin Li, Xiaoqing Xie, Fuli Ma, Dan Wang, Lan Wang, Jianxia Zhang, Yan Xu, Xiping Wang, Chaohong Zhang, Yuejin Wang

**Affiliations:** 1 College of Horticulture, Northwest A&F University, Yangling, Shaanxi 712100 The People’s Republic of China; 2 Key Laboratory of Horticultural Plant Biology and Germplasm Innovation in Northwest China, Ministry of Agriculture, Yangling, Shaanxi 712100 The People’s Republic of China; 3 State Key Laboratory of Crop Stress Biology in Arid Areas, Northwest A&F University, Yangling, Shaanxi 712100 The People’s Republic of China

## Abstract

Attention has become focused on resveratrol not only because of its role in grapevine fungal resistance but also because of its benefits in human health. This report describes the Chinese wild grapevine *Vitis quinquangularis* accession Danfeng-2 in relation to the high resveratrol content of its ripe berries. In this study, we used isobaric tags for relative and absolute quantification (iTRAQ) tandem mass spectrometry strategy to quantify and identify proteome changes, resulting in the detection of a total of 3,751 proteins produced under natural conditions. Among the proteins quantified, a total of 578 differentially expressed proteins were detected between Danfeng-2 and Cabernet Sauvignon during berry development. Differentially expressed proteins are involved in secondary metabolism, biotic stress, abiotic stress and transport activity and indicate novel biological processes in Chinese wild grapevine. Eleven proteins involved in phenylpropanoid metabolism and stilbene synthesis were differently expressed between Danfeng-2 and Cabernet Sauvignon at the veraison stage of berry development. These findings suggest that Chinese wild *V. quinquangularis accession* Danfeng-2 is an extremely important genetic resource for grape breeding and especially for increasing the resveratrol content of European grape cultivars for disease resistance and for improved human nutritional benefits.

## Introduction

Grapes, *Vitis* spp, can be eaten fresh or processed into wine, raisins and a range of other foods. They are thus one of the world’s most widely-cultivated fruit crops and are the basis of an age-old industry. This industry is exceedingly large and has expanded very rapidly over the last 50 years. The European grapevine (*Vitis vinifera* L.) is the predominant species grown, mainly because it is highly valued for its winemaking properties. There are a very large number of named *V. vinifera* cultivars, some ancient and some modern. However, most of these *V. vinifera* cultivars are very susceptible to fungal pathogens, which cause major economic loss. Disease management comes with its costs. These costs are not only financial (loss of yield and expense of control) but also environmental (impacts on wildlife and heavy-metal residues), and so pose a risk both to this and to future generations^1^.

Intraspecific grape breeding for increased disease resistance is one solution, but disease resistance within *V. vinifera* is quite limited. Instead, interspecific breeding to produce hybrid cultivars offers a much more promising solution. Either way, disease-resistant germplasm is essential. In general, the wild *Vitis* species offer a very useful source of pathogen resistance^2^. China is one of three main centers of origin of *Vitis*, where a large number of wild *Vitis* species are conserved. The Chinese wild *Vitis* spp. represent an important part of the world *Vitis* germplasm resource with good pathogen resistance and high levels of resveratrol^3, 4^. Numerous Chinese wild grape accessions are highly disease-resistant, among these are *V. pseudoreticulata* ‘Baihe-35-1’ and *V. quinquangularis* ‘Danfeng-2’^3^.

Resveratrol, which is well known as a phytoalexin, is considered to be associated with the grapevine’s resistance to fungi^5^. In the past few years, increasing attention has been paid to grape germplasm that is high in resveratrol. In human medicine, resveratrol has been shown to possess anti-inflammatory, anti-platelet, anti-carcinogenic, antifungal and antibacterial activities^6^. Stilbene synthase is the key enzyme for resveratrol synthesis^7^. Stilbenes are strongly accumulated in grapevine when stilbene synthase is overexpressed and disease resistance of transgenic grapevines has been significantly increased^6^. Indeed, transformation of stilbene synthase genes in tobacco^8^, rice^9^, alfalfa^10^, kiwifruit^11^, wheat^12, 13^, apple^14^, papaya^15^, poplar^16^, Arabidopsis^17^, pea^18^ and hops^19^ can enhance disease resistance or raise the resveratrol content of the receptor plants.

Danfeng-2, a Chinese wild *V. quinquangularis* accession, has been confirmed in a number of studies to contain high levels of resveratrol^20, 21^. In a previous study, we have isolated 41 stilbene synthase genes from *V. quinquangularis* Danfeng-2 and compared these with the expression profiles of the stilbene synthase gene family in *V. vinifera* cv. Pinot Noir during *Uncinula necator* infection^20^. Soon after infection, a fruit-specific, highly-expressed stilbene synthase gene *VqSTS6* from *V. quinquangularis* Danfeng-2 was transformed to *V. vinifera* cv. Thompson Seedless and high levels of *Trans*-resveratrol were detected in the leaves of the transgenic lines^22^. However, the mechanism for high levels of resveratrol in *V. quinquangularis* Danfeng-2 is still unknown.

A number of molecular biology tools have been developed to study a range of biological problems. Proteomics has been proved an effective tool to analyze the proteome changes in grape under abiotic and biotic stress as well as during berry development^23–27^. Dimensional electrophoresis followed by Matrix-Assisted Laser Desorption/Ionization Time of Flight Mass Spectrometry (MALDI-TOFI-MS) has been used to characterize the differential expressed proteins in *V. vinifera* ^28–31^. However, isobaric tags for relative and absolute quantification (iTRAQ) can determine accurate protein level changes and more protein differences than gel-based proteomics. In recent years, more researchers have used iTRAQ followed by liquid chromatography-mass spectrometry/mass spectrometry (LC-MS/MS) to carry out proteome study in *Vitis* and iTRAQ has proved to be an excellent method for studying biological problems in grapevine^32–35^.

In this study, the Chinese wild species *V. quinquangularis* Danfeng-2 and *V. vinifera* cv. Cabernet Sauvignon, a very well-known winemaking grape cultivar, were analyzed by iTRAQ to obtain specific protein identification and for future use in improving grapevine breeding properties. The aim was to obtain and identify specific proteins expressed from *V. quinquangularis* accession Danfeng-2 with high levels of resveratrol and disease resistance during the various stages of berry development. Additionally, differentially expressed proteins involved in secondary metabolism, biotic stress, abiotic stress and transport activity will be valuable for revealing the elite traits of high resveratrol content and disease resistance from Chinese wild *Vitis quinquangularis* Danfeng-2. The levels of selected proteins were correlated with their transcript levels by quantitative real-time polymerase chain reaction (RT-PCR) analysis. The novel findings of this study will provide new opportunities to explore the mechanisms involved in the high levels of resveratrol and disease resistance found in Chinese wild *V. quinquangularis*. This study also proposes candidate genetic resource for further grape breeding, designed to increase the resveratrol content of some of the more popular wine grape cultivars.

## Results

### iTRAQ analyses of proteins in berries of *V. quinquangularis* accession Danfeng-2 and *V. vinifera* cv. Cabernet Sauvignon at four developmental stages

**Figure 1:**
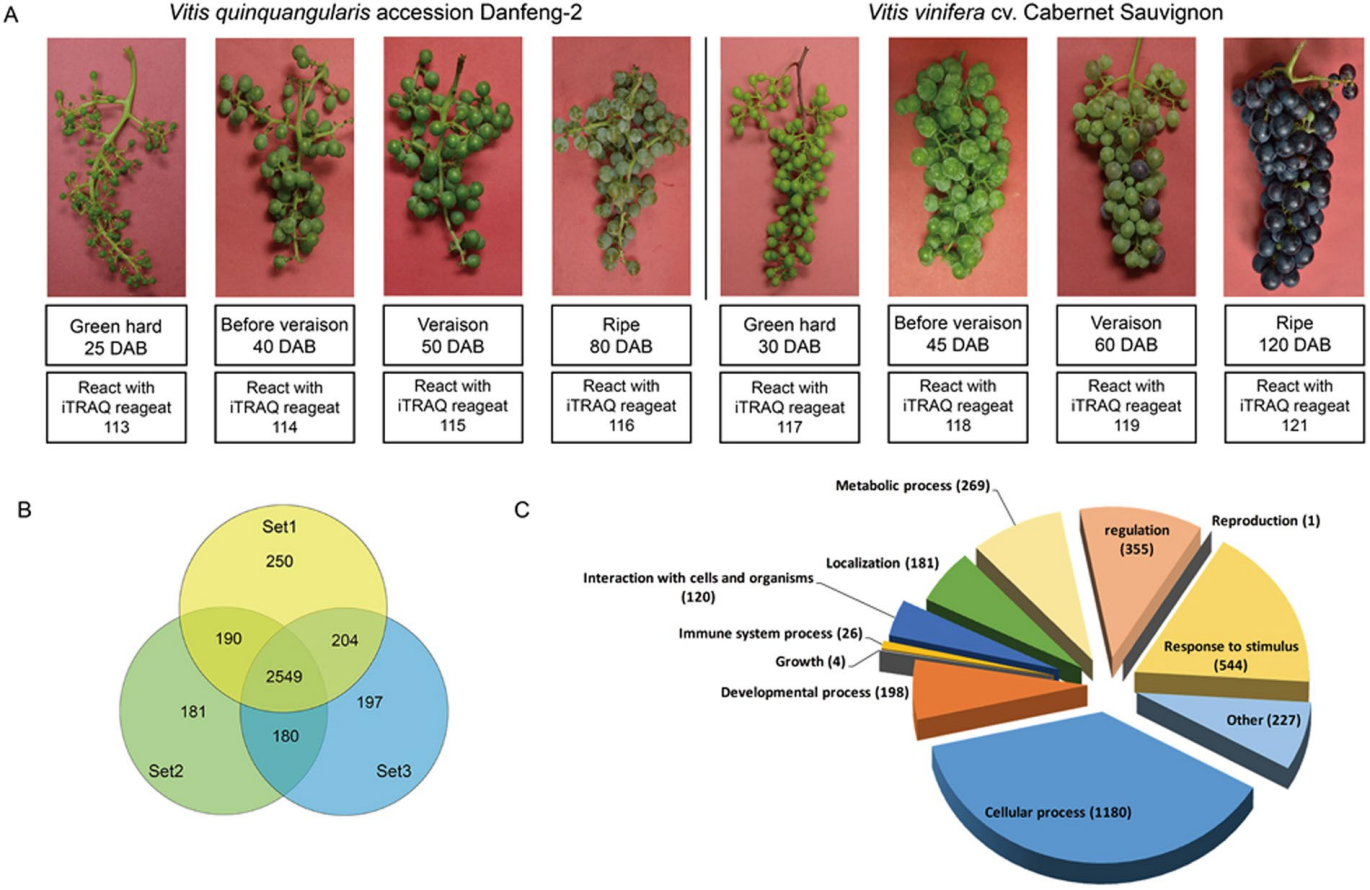
Workflow and proteins identified in four developmental stages of berries of *Vitis quinquangularis* accession Danfeng-2 and control grapevine *V. vinifera* cv. Cabernet Sauvignon with iTRAQ. (**A**) iTRAQ 8-plex labeling of different developmental stages (green hard, before veraison, veraison and ripe). DAB, days after blooming; (**B**) Venn diagram showing proteins among the three iTRAQ data sets of Danfeng-2 and Cabernet Sauvignon; Set1, Set2 and Set3 represented three independent biological replicates. (**C**) GO term enriched broader functional classification of 3751 proteins identified during four developmental stages of berries of Danfeng-2 and Cabernet Sauvignon. Number of each GO term was next to the corresponding pie.

A flow chart for the iTRAQ analysis is shown in Fig. 1A. The samples were labeled with different iTRAQ reagents (Sigma-Aldrich, Germany) and mixed for MS/MS analysis. The raw data were then analyzed using MASCOT, and the proteins were quantified. For three iTRAQ 8 × plex experiments, totals of 3,193, 3,100 and 3,130 proteins were identified, respectively. Amongst these, 2549 of the proteins generated were common. A Venn diagram shows the details of the overlap, as well as the proteins that were unique to each replicate (Fig. 1B). A total of 3,751 proteins that were unique to a single replicate were identified and the GO terms (biological process) of the proteins identified were clustered (Fig. 1C). The majority of the proteins were involved in cellular processes (38%), responses to stress (18%), and regulation (11%). Details of the Uniport IDs, percent coverage, number of unique peptides, ratios of different plex, protein length, predicted molecular weights and isoelectric points are listed in Supplementary Tables S2 and S3. Of the proteins identified, we generated many that are involved in secondary metabolism and stress responses. Of the proteins related to secondary metabolism, phenylalanine ammonialyase, chalcone synthase, resveratrol glucosyltransferase (RSGT), flavonoid synthesis proteins, and anthocyanin synthesis proteins were identified and verified. Of the stress response proteins, we annotated alcohol dehydrogenase proteins, major latex proteins (MLP), ubiquitin proteins, the mildew resistance locus o (MLO) proteins, pathogenesis-related proteins, aquaporin, heat shock proteins, calcineurin B-like proteins, pectinesterases, dehydrins, EF-hand calcium binding proteins and phospholipases and others.

### Contents of resveratrol, piceid, flavonoid and anthocyanin in berries of *V. quinquangularis* accession Danfeng-2 and *V. vinifera* cv. Cabernet Sauvignon

**Figure 2:**
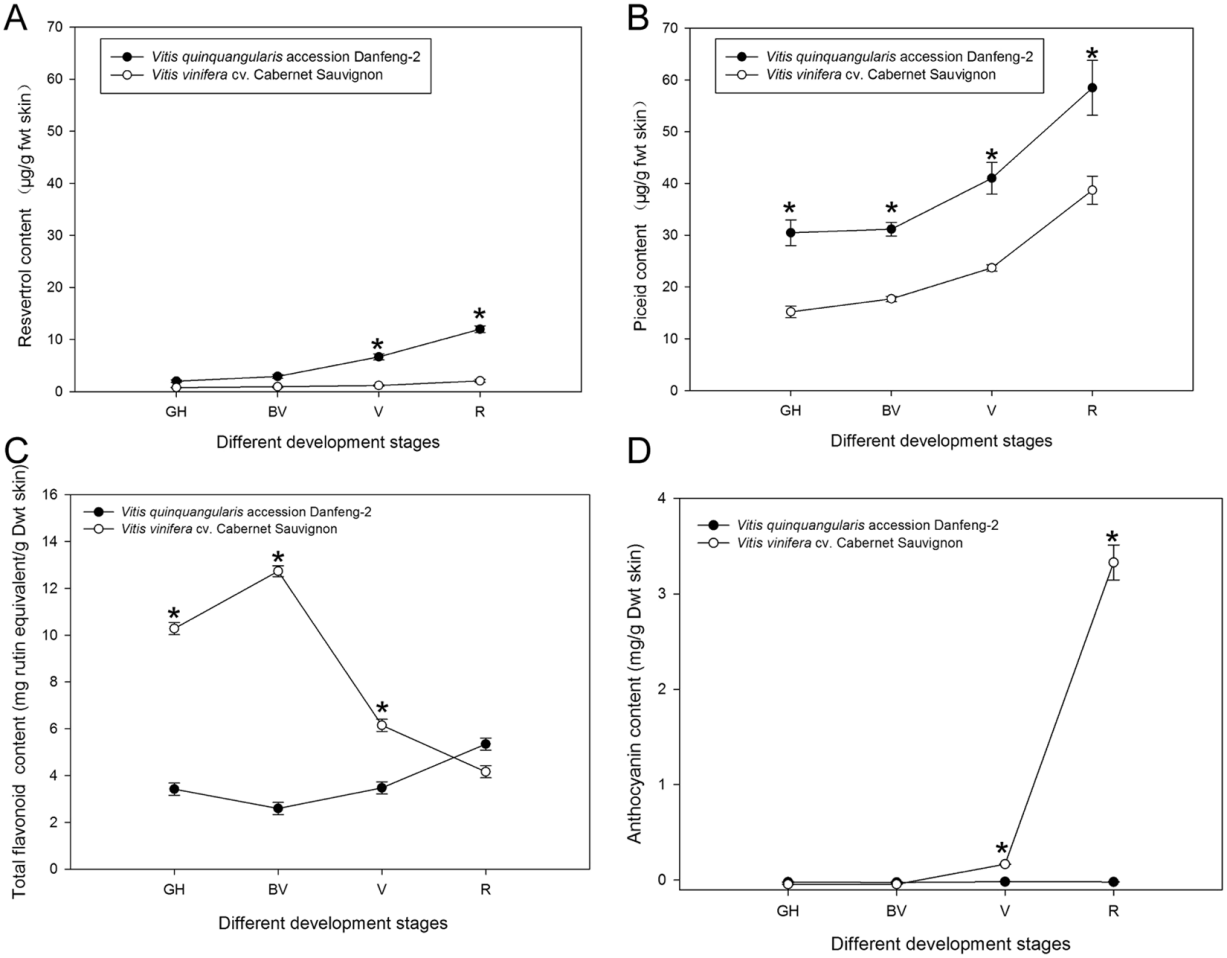
Determination of metabolites in berry skins of *Vitis quinquangularis* accession Danfeng-2 and *V. vinifera* cv. Cabernet Sauvignon at four different development stages. GH, green hard, BV, before veraison, V, veraison, and R, ripe. (**A**) Trans-resveratrol content; (**B**) Piceid content. (**C**) Total flavonoid content; (**D**) Anthocyanin content. The *p*-value level less than 0.05 was considered significantly different at each development stage between *Vitis quinquangularis* accession Danfeng-2 and *V. vinifera* cv. Cabernet Sauvignon and labeled with an asterisk.

Danfeng-2 believed to contain high amounts of resveratrol. We determined the contents of resveratrol and piceid using HPLC and results indicate significant differences in trans-resveratrol and piceid between Danfeng-2 and Cabernet Sauvignon (Fig. 2A and B). The resveratrol content of Danfeng-2 berries increased continually during ripening and was 5.70-times greater than in Cabernet Sauvignon at stage of veraison and 5.79-times greater at stage of ripe. The content of piceid was at least 1.50-times higher in Danfeng-2 than in Cabernet Sauvignon. The main stilbene components in Danfeng-2 were significantly higher than in Cabernet Sauvignon. To compare the differences in flavonoid and anthocyanin accumulation between Danfeng-2 and Cabernet Sauvignon, we measured the contents of total flavonoid and total anthocyanins. Total flavonoids in Danfeng-2 berry skins first declined then increased (Fig. 2C). However, total flavonoids in Danfeng-2 were significantly less than in Cabernet Sauvignon during development except at ripe stage (Fig. 2C). Anthocyanin accumulation was not detected in the berry skins of Danfeng-2, but in Cabernet Sauvignon anthocyanin content began to rise at veraison stage (Fig. 2D).

### Differentially expressed proteins between *V. quinquangularis* accession Danfeng-2 and *V. vinifera* cv. Cabernet Sauvignon during berry development

**Figure 3:**
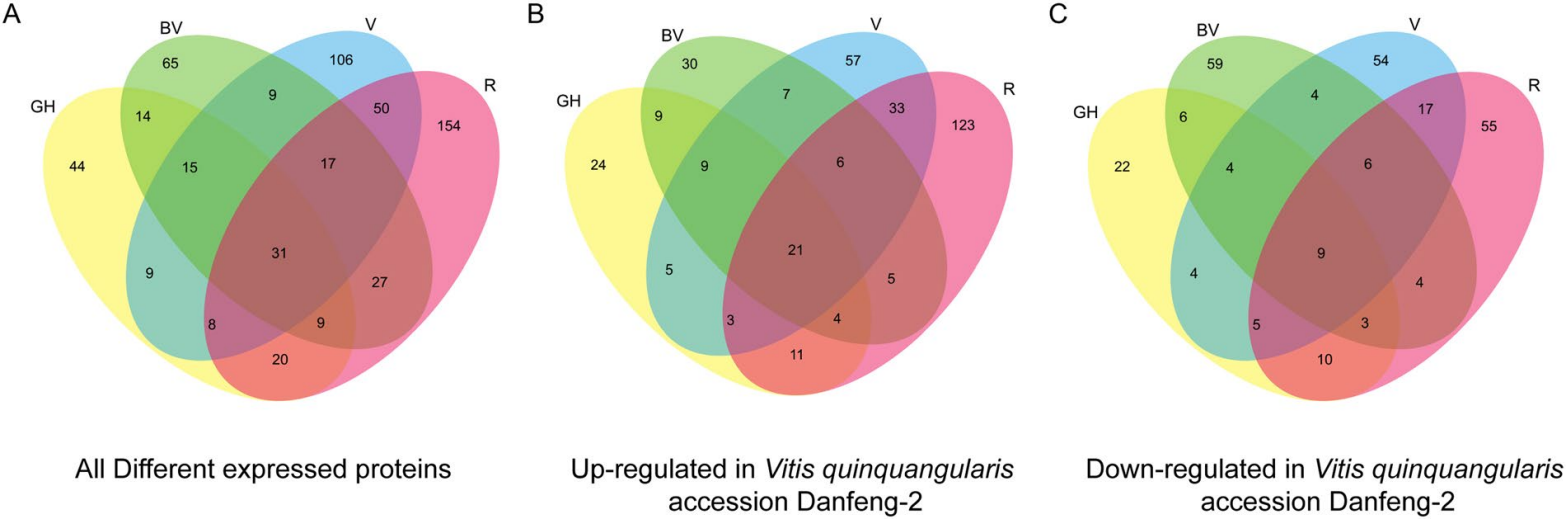
Overlaps of different expression proteins over the developmental stages between *Vitis quinquangularis* accession Danfeng-2 and *V. vinifera* cv. Cabernet Sauvignon. GH, green hard, BV, before veraison, V, veraison, and R, ripe. (**A**) Venn diagram illustrating overlaps of 578 differential proteins at all stages between Danfeng-2 and Cabernet Sauvignon. DEPs-differentially expressed proteins; (**B**) Venn diagram illustrating overlaps of proteins with higher expression profiles in Danfeng-2 at each stage; (**C**) Venn diagram illustrating overlaps of proteins with lower expression profiles in Danfeng-2 at each stage.

We identified proteins that were differentially expressed during development in the two grape genotypes. Comparing the four stages of berry development, proteins with expression level differences >1.5-fold were considered to have been up-regulated and those <0.67-fold to have been down-regulated. The differentially expressed proteins between Danfeng-2 and Cabernet Sauvignon at each of the four developmental stages were identified. There were 151 differentially expressed proteins identified for stage GH, 188 for stage BV, 246 for V and 317 for R (Fig. 3A, Supplementary Table S4). Up-regulated proteins in Danfeng-2 were 87 for GH, 92 for BV, 142 for V and 208 for R (Fig. 3B). The up-regulated proteins in Cabernet Sauvignon were 64 for GH, 96 BV, 104 for V and 109 for R (Fig. 3C). The differentially expressed proteins were involved in various cellular processes and annotation of differentially expressed proteins was carried out using the UniProt database.

### Proteomic analysis of the grape epicarp of Danfeng-2 with Cabernet Sauvignon as control, reveals distinct developmental processes

**Figure 4:**
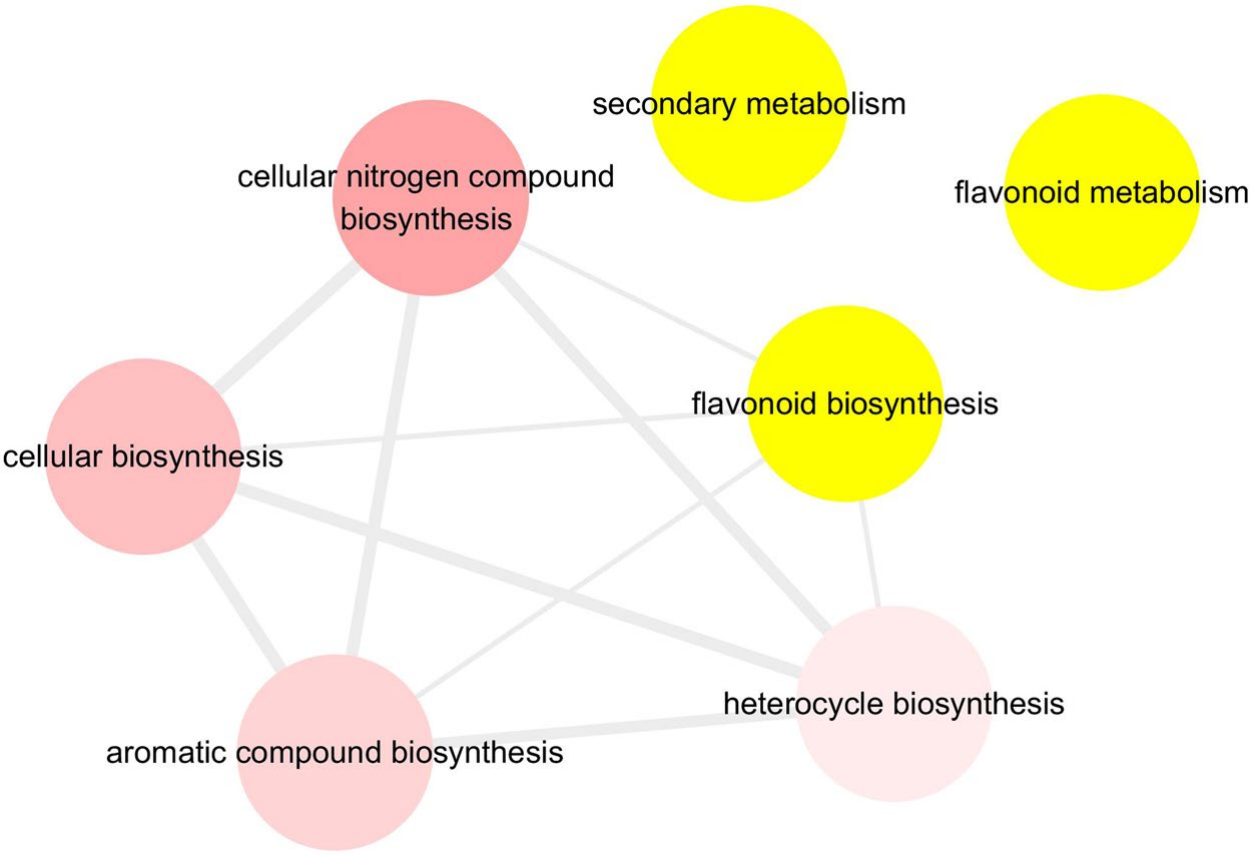
Enriched GO terms of the different expression proteins related with secondary metabolism over the developmental stages of *Vitis quinquangularis* accession Danfeng-2 and *V. vinifera* cv. Cabernet Sauvignon.

The iTRAQ analysis identified differentially expressed proteins in Danfeng-2 and Cabernet Sauvignon at each of the four developmental stages as described above. GO annotated was based on the annotation in the Uniport database. To visualize the differential proteins, the *p-value* of the GO terms were enriched in agriGO and displayed using Cytoscape (Fig. 4, Supplementary Table S5). Enriched GO terms were clustered, based on their functions. Most of the differentially expressed proteins were annotated to be cellular ketone metabolism and rRNA metabolism. However, differentially expressed proteins involved in secondary metabolic pathways were also identified. GO terms related to secondary metabolic were highlighted with yellow background (Fig. 4). These results indicated many differentially expressed proteins are involved in flavonoid metabolism. In addition, flavonoid biosynthesis was related with heterocyclic biosynthesis, aromatic compound biosynthesis, cellular biosynthesis, and cellular nitrogen compound biosynthesis (Fig. 4).

### Proteins involved in the phenylalanine metabolism pathway

**Figure 5:**
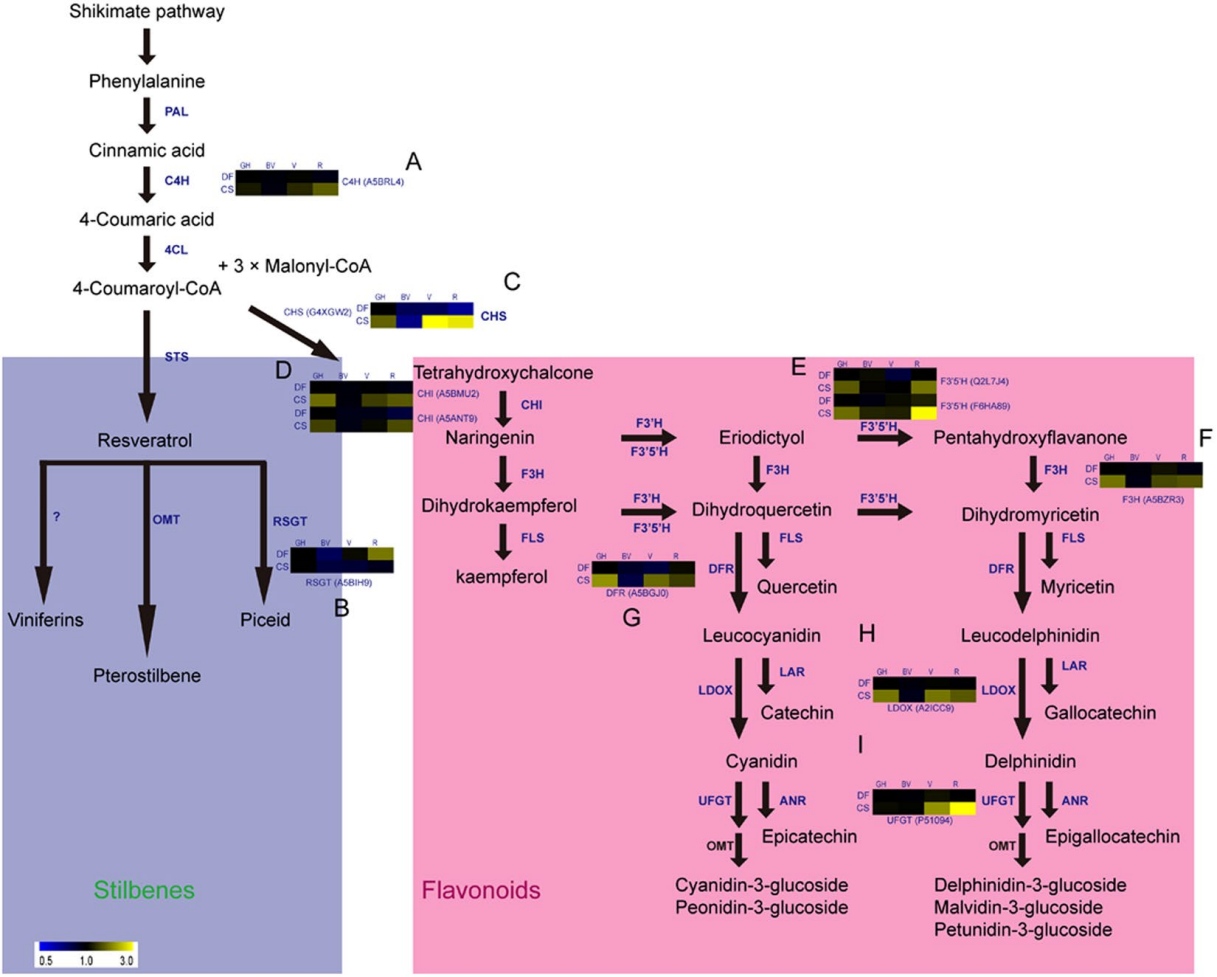
Differential expressed proteins involved in phenylalanine metabolism pathway revealed specific protein promoting stilbenes accumulation in *V. quinquangularis* accession Danfeng-2. (**A**) C4H, Resveratrol metabolism pathway, (**B**) RSGT, and flavonoid metabolism (**C**) CHS, (**D**) CHI, (**E**) F3′5′H, (**F**) F3H, (**G**) DFR, (**H**) LDOX, and (**I**) UFGT, were identified. Especially, a resveratrol glucosyltransferase (A5BIH9) was significantly up-regulated at stage Ripe in Danfeng-2. PAL, phenylalanine ammonia-lyase, C4H, trans-cinnamate 4-monooxyygenase, 4CL, 4-coumarate-CoA ligase, STS, stilbene synthase; RSGT, resveratrol glucosyltransferase, OMT, O-methyltransferase, CHS, chalcone synthase, CHI, chalcone isomerase, F3H, flavanone 3-hydroxylase, F3′H, flavonoid 3′-hydroxylase; F3′5′H, flavonoid 3′,5′-hydroxylase; DFR, dihydroflavonol-4-reductase; FLS, flavonol synthase, LDOX, leucoanthocyanidin dioxygenase, LAR, leucoanthocyanidin reductase, ANR, anthocyanidin reductase, UFGT, UDP-glucose:flavonoid 3-O-glucosyltransferase. Heat maps of proteins were constructed with iTRAQ-derived quantitative data. D-2, Danfeng-2, CS, Cabernet Sauvignon. GH, green hard, BV, before veraison, V, veraison, and R, ripe. The proteins that increased and decreased are displayed in yellow and blue, respectively.

Differential expressed proteins involved in the phenylalanine metabolism pathway revealed specific proteins promoting stilbene accumulation in *V. quinquangularis* accession Danfeng-2. To generate a more complete picture of the dynamic changes in protein levels of enzymes involved in the phenylalanine metabolic pathway during development, their expression profiles were examined in more detail. The phenylalanine metabolic pathway is a fully characterized metabolic pathway in plants, including in grapevine. A specific STS branch has evolved that results in the accumulation of the polyphenol, resveratrol. Here, the expression profiles of several enzymes in this pathway showed significant differences in abundance in the two varieties, at the veraison and ripe stages. Cabernet Sauvignon berries began to accumulate color at the veraison stage and the enzymes in the CHS branch showed several-fold higher expression levels than the corresponding enzymes in Danfeng-2, while the distinction at the ripe stage was less obvious. The proteins identified with significant differences in expression in relation to the phenylalanine metabolic pathway are shown in Fig. 5. Proteins in the pathway downstream of CHS began to accumulate at the veraison stage in Cabernet Sauvignon, while no differences were observed in Danfeng-2. Trans-cinnamate 4-monooxyygenase (C4H) (A5BRL4), an enzyme downstream of phenylalnine ammonialyase, expressed more highly in Cabernet Sauvignon than in Danfeng-2 at the ripe stage (Fig. 5A). A resveratrol glucosyltransferase (RSGT) (A5BIH9) accumulated to high levels in Danfeng-2 at the ripe stage, which was not observed in Cabernet Sauvignon (Fig. 5B). However, the enzymes in the CHS branch in Cabernet Sauvignon showed significantly higher levels than that in Danfeng-2 including CHS (Fig. 5C), CHI (Fig. 5D), F3′5′H (Fig. 5E), F3H (Fig. 5F), DFR (Fig. 5G), LDOX (Fig. 5H), UFGT (Fig. 5I) at the ripe stage and/or at the veraison stage and green hard stage. These enzymes control the synthesis of flavonoid and anthocyanins. Next we used RT-PCR to gain support for the protein expression profiles. A possible explanation for the interesting resveratrol accumulation pattern in Cabernet Sauvignon is that STS and CHS compete during berry development for the common substrates, p-coumaroyl-CoA and malonyl-CoA. The expressions of several genes related to the pathway, such as *RSGT*, *STS*, *MYB14, MYB15*, *MYB5a*, *MYB5b*, *MYBPA1* and *MYBA1* were analyzed using qRT-PCR (Fig. 5A–H). The results demonstrate that these genes are expressed differently for same development stage. The expression of gene *RSGT* gradually increased during berry development in Danfeng-2 but decreased in Cabernet Sauvignon. Meanwhile, *RSGT* was highly expressed at ripe stage (Fig. 5A). The expression of STS reached a peak in Danfeng-2 at the veraison stage when it was 2.6-fold greater than in Cabernet Sauvignon (Fig. 5B). However, it was 2.3-fold more highly expressed in Cabernet Sauvignon than in Danfeng-2 at the ripe stage (Fig. 5B). The *MYB14* was more highly expressed in Danfeng-2 at all four stages (Fig. 5C). The transcript levels of *MYB15* in Danfeng-2 were 4.3-fold higher than in Cabernet Sauvignon at stage veraison and 8.5-fold higher at stage ripe (Fig. 5D). The genes *MYB5a*, *MYB5b*, *MYBPA1* and *MYBPA1* are related to anthocyanins and flavonoids, and regulate *CHS*, *chalcone isomerase*, *leucoanthocyanidin dioxygenase* and *anthocyanin reductase* in the phenylalanine metabolic pathway. Surprisingly, transcript expression levels of *MYBPA1* were 6.4-fold higher in Cabernet Sauvignon than in Danfeng-2 at Stage veraison (Fig. 5G). *MYBA1* was not expressed in Danfeng-2 and expressed highly in Cabernet Sauvignon at stages veraison and ripe (Fig. 5H).

### Evaluation of the transcript levels corresponding to the differentially expressed proteins

**Figure 6:**
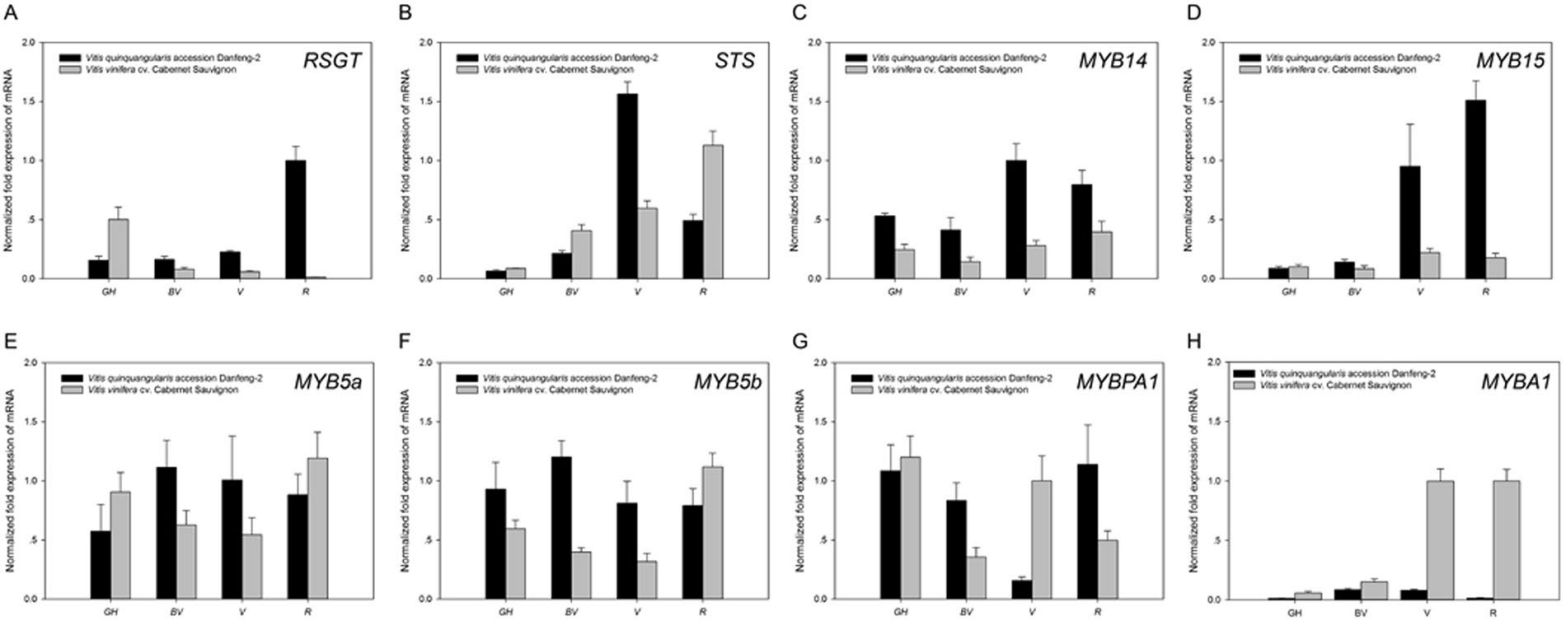
Relative expression levels of related transcripts. Data were normalized with the housekeeping gene GAPDH. Genes involved in stilbene synthesis are (**A**) *RSGT*; (**B**) *STS*; (**C**) *MYB14*; (**D**) *MYB15*; Genes involved in flavonoid and anthocyanin synthesis are (**E**) *MYB5a*; (**F**) *MYB5b*; (**G**) *MYBPA1*; (**H**) *MYBA1*. 2^−ΔΔCt^ method was used to calculate the relative expression for each gene. Each value represents the means ± SE of three different experiments.

**Figure 7:**
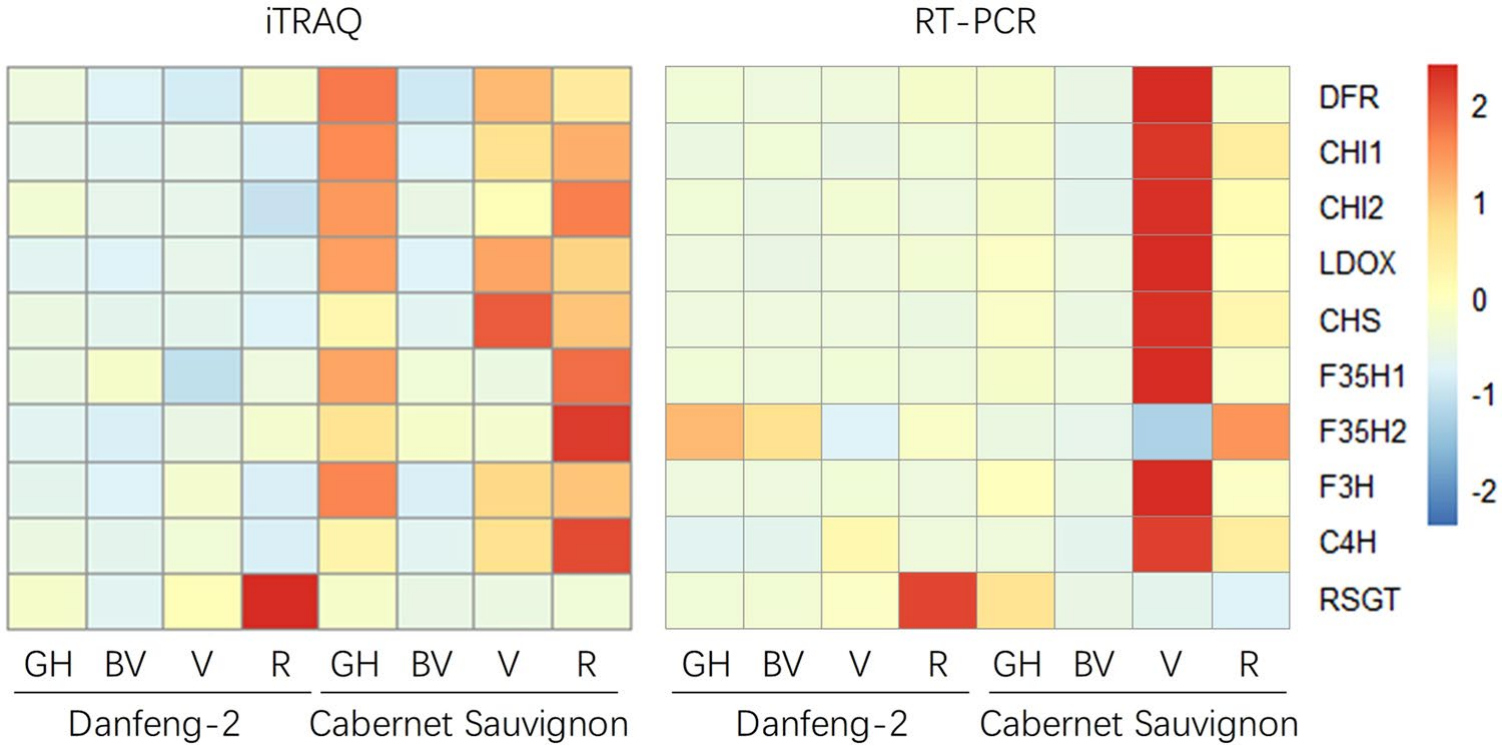
Correlation of protein and mRNA levels of stilbene and flavonoid biosynthesis related members. C4H, trans-cinnamate 4-monooxyygenase, RSGT, resveratrol glucosyltransferase, CHS, chalcone synthase, CHI, chalcone isomerase, F3H, flavanone 3-hydroxylase; F3′5′H, flavonoid 3′,5′-hydroxylase; DFR, dihydroflavonol-4-reductase; LDOX, leucoanthocyanidin dioxygenase. GH, green hard, BV, before veraison, V, veraison, and R, ripe. The proteins that increased and decreased are displayed in red and blue, respectively.

The details of the iTRAQ 8-plex data set and mRNA expression data are listed in Supplementary Table S6. Genes involved in the phenylalanine metabolic pathway were preferentially chosen for qRT-PCR analysis to complement the proteomic data. The targeted genes/proteins were a dihydroflavonol-4-reductase (A5BGJ0), a chalcone isomerase 1 (A5BMU2) and a chalcone isomerase 2 (A5ANT9), a leucoanthocyanin dioxygenase (A2ICC9), a CHS (G4XGW2), a flavonoid 3′,5′-hydroxylase 1 (Q2L7J4) and a flavonoid 3′,5′-hydroxylase 2 (F6HA89), a flavanone 3-hydroxylase (A5BZR3), a cinnamate-4-hydroxylase (A5BRL4), a resveratrol glucosyltransferase (A5BIH9), a ubiquitin conjugating enzyme 32 (A5BBF4), a leucine-rich repeat protein (A5BFQ1), a glycine rich protein (A5AI47), a plasma membrane intrinsic protein 2;3 (A3FA68), and a universal stress protein (D7TA35). The correlations between the detected proteins and their trancript levels were not strictly linear but generally showed similar trends. One notable discrepancy was the glycine rich protein (A5AI47), which displayed the highest trancript levels at stage V but the highest abundance of protein accumulation at stage R in Danfeng-2. Another was the mRNA levels of CHS (G4XGW2), which was up-regulated over 1,000-fold between stage BV and V but the protein levels increased only 5.4-fold. Stilbene and flavonoid biosynthesis related proteins were selected for correlation analysis with their responsive transcript levels (Fig. 6). The expression trends between protein and mRNA were similar although most proteins had highest expression profile in ripe stage while mRNA was highest in the veraison stage of Cabernet Sauvignon. The related coefficient of protein levels and transcript levels were 0.452 (Fig. 7).

## Discussion

Previous reports have made significant advances in understanding the proteome changes during grape berry ripening and the overall events in berry development are now generally understood^23, 27, 36^. Proteins related to photosynthesis and metabolism are highly expressed at the beginning of berry development^23, 27, 36^, while defense proteins are highly accumulated at harvest^23^. A comparative proteomic analysis based on cross-progeny populations of red and white *V. vinifera* grapes^37^ indicates many differences occur before veraison. However, comparison of proteomes between Chinese wild species and *V. vinifera* cultivars have rarely been reported. The mechanisms associated with the valuable traits of the wild species still need to be determined. The present study demonstrates the dynamic changes of proteins at different development stages between *V. quinquangularis* Danfeng-2 and *V. vinifera* cv. Cabernet Sauvignon. It also identifies candidate proteins related to resveratrol synthesis.

Stress response proteins are crucial for plant resistance to biotic and abiotic stresses^38, 39^. Of the differentially expressed stress response proteins between Danfeng-2 and Cabernet Sauvignon, we found a number of biological processes were involved. The up-regulated proteins in Danfeng-2 was mainly related to energy metabolism. Proteins involved in photosynthesis with high expression in Danfeng-2 suggests its berries collect more energy for production of down-stream products including secondary metabolites. Sedoheptulose-1,7-bisphosphatase can enhance photosynthesis and growth^40^. In addition, chlorophyll a-b binding proteins are responsible for harvesting light^41^. Other stress related proteins were also detected, such as dehydrins, heat shock proteins, and defensing. These indicate powerful stress-resistance in the Chinese wild grape. However, the up-regulated proteins in Cabernet Sauvignon belonged to a distinct category. The MLP-like proteins are believed to improve biotic and abiotic resistance in Arabidopsis^42^. Pathogenesis-related protein PR-4 and serine/threonine-protein kinase were also detected. The different categories of proteins between Danfeng-2 and Cabernet Sauvignon reveal different accumulations of stress proteins in Chinese wild grape during berry development. The chlorophyll concentrations in grape berries and transcripts of chlorophyll a-b binding proteins decrease during ripening^43^. This could explain the high level of disease resistance of the Chinese wild grape species.

The phenylalanine metabolic pathway, is one of the best characterized metabolic pathway in plants. It produces a variety of secondary metabolites including flavonoids, lignins and taxon-specific compounds, such as the stilbenes in grapevine^44^. Among the metabolites, flavonoids and stilbenes share the same precursors, namely, p-coumaroyl-CoA and malonyl-CoA^45^. In our search of the Uniprot KB database, the functions of thousands of proteins are annotated, However, we did not generate the expression of stilbene synthase. The reason may be that the protein level of stilbene synthase was below the minimum threshold of iTRAQ. Several previous studies have also failed to identify this protein^26, 46^. Fortunately, we identified a homolog (A5BIH9) of resveratrol glucosyltransferase (RSGT), a protein that makes the stilbenoid glucoside from resveratrol^47^. RSGT is up-regulated in Danfeng-2 at the R stage (Fig. 5B). The Q-RT PCR result for RSGT agreed with the iTRAQ one (Fig. 5A). This glucosyltransferase follows stilbene synthase and may control the carbon flow to stilbenes but this must still be confirmed. In *V. vinifera* cv. Muscat Hamburg berries, twelve *STS* genes and two *phenylalanine ammonia lyase* (*PAL*) genes have been observed to be up-regulated during berry ripening^48^. Also, the phenylpropanoid/stilbene biosynthetic genes *PAL*, *CHS* and *STS* genes have been reported to be highly expressed in ripe Shiraz berries^49^. So the transcript level of stilbene synthases and the positive regulation transcript factor of stilbene synthases *MYB14* and *MYB15* ^50^, were also examined. *STS* showed the same expression profile as that described above for Cabernet Sauvignon, while its expression peaked at stage V in Danfeng-2 (Fig. 5B). The transcript levels of both *MYB14* and *MYB15* were higher in Danfeng-2 at stage V and R (Fig. 5C and D). Thus, high resveratrol content in grapevine seems to be related to these genes.

Major differences in the protein profiles involved in the flavonoid metabolic pathway show obvious increases from BV to V in Cabernet Sauvignon. CHS, CHI, F3H, F3′,5′H and UFGT, which control carbon flux along the CHS pathway, were identified. Our protein expression results could help explain the carbon flow when compared with the levels of total anthocyanin and flavonoid (Fig. 5). Transcript factors such as *MYB5a*, *MYB5b* and *MYBPA1*, control the expressions of enzymes in the flavonoid metabolic pathway^51^, however, the transcript levels of these were not significantly elevated in Cabernet Sauvignon (Fig. 5E and G). Although we detected the expression profile of these well studied transcript factors, our results indicated there are other regulators that control the flavonoid metabolic pathway. The high protein levels of enzymes in the flavonoid metabolic pathway (e.g. CHS, CHI, F3H, F3′5′H) (Supplementary Table S6), suggests complex processes exist in the berries between transcript factors and their target genes. *MYBA1* which regulates the key enzyme UFGT for anthocyanins^51^, was not expressed at any developmental stage in Danfeng-2 but it was highly expressed in Cabernet Sauvignon at stages V and R (Fig. 5H). These results suggest there are huge differences between red and white grapevine cultivars when berries start to color.

Our earlier studies have systematically characterized stilbene synthase gene function in Chinese wild grape and their genetic transformation to *V. vinifera* ^2, 22, 52^. There were higher expression levels of stilbene synthase genes in the skins of berries of *V. quinquangularis* accession Danfeng-2 than in those of *V. vinifera* cv. Pinot Noir. Conversely, the resveratrol content of Danfeng-2 berries is higher than that of in Pinot Noir and Cabernet Sauvignon berries (Shi *et al*., 2014), which agrees with our results. Furthermore, the results for *V. pseudoreticulata* accession Baihe35-1 showed that the promoter activity of the stilbene synthase gene was strongly regulated by both pathogen and abiotic stresses^52, 53^. Also, transient expression of the stilbene synthase gene in tobacco increased the resveratrol content. When stilbene synthase genes were stably transformed into *V. vinifera*, the level of stilbenes and disease tolerance were both increased significantly^22^. Here, an enzyme RSGT, which glycosylates resveratrol into the more stable piceid, was highly expressed in Danfeng-2 (Fig. 5). Based on all the above results, we propose a potential mechanism to explain the high resveratrol content in the Chinese wild grapevines.

Our study provides a comparison of the proteomes of the Chinese wild grape *V. quinquangularis* and *V. vinifera*. Our results provide new information on the mechanisms involved in resveratrol accumulation and the defense systems of grapevines. Moreover, it becomes clear that Chinese wild grape germplasm will in time be able to contribute to the development of disease resistant cultivars of *V. vinifera* and hence benefit both the grape industry and the health benefits associated with grapes and grape products. This study analyzed specific proteins and elucidated the main features of the Danfeng-2 proteome during berry development. The two proteins, glucosyltransferase (RSGT) and stilbene synthase (STS), are involved in the phenylalanine metabolic pathway. These were found to be increased and in Chinese wild *V. quinquangularis accession* Danfeng-2 were apparently responsible for the accumulation of resveratrol and for known and significant changes in specific metabolites. The candidate proteins and transcription factor genes related to the high resveratrol content of Chinese wild *V. quinquangularis* are identified. The specific germplasm Chinese wild grape Danfeng-2 will likely be very useful for grape breeding to increase the resveratrol content of the European grapevine cultivars which will increase both the economics of grape production and the health benefits associated with human consumption of grape products.

## Materials and Methods

### Plant material

Grape berries of *V. quinquangularis* accession Danfeng-2 were sampled from the vineyard of Northwest A&F University, China in 2013 and *V. vinifera* cv. Cabernet Sauvignon was used as control. Both *V. quinquangularis* accession Danfeng-2 and *V. vinifera* cv. Cabernet Sauvignon were well watered and grown in a reasonable row spacing with no pest and disease occurred. Grape Cluster bagging after fruit set was conducted to avoid abiotic and biotic stresses. Berries were collected at stages spanning the developmental period from fruit set to harvest ripe. The berry stages for Cabernet Sauvignon berries were classified according to the system described by Lorenz *et al*.^54^. Danfeng-2 is one of most important wild grape germplasm resources. It comes from the mountainous regions of Danfeng county, Shannxi province, and has white berries and its phenophases (emergence of leaves, flowers and berries) are somewhat later than those of Cabernet Sauvignon^20, 55^. We classified Danfeng-2 berries on the basis of a previously-determined growth curve of fruit weight. The Danfeng-2 samples were collected from four developmental stages: green hard (GH), 25 days after bloom (DAB); before veraison (BF), 40 DAB; veraison (V), 50 DAB; ripe (R), 80 DAB. Meanwhile, the Cabernet Sauvignon samples were collected at the same developmental stages: GH, 30 DAB; BF, 45 DAB; V, 60 DAB; R, 110 DAB. Biological replicate berries at the same developmental stages were selected from different clusters. After detaching a berry, the epicarp was carefully separated using a sharp scalpel and forceps, and then frozen in liquid nitrogen and stored at −80 °C pending analysis^21^.

### Protein extraction

Protein extraction used the method described by Kambiranda, *et al*.^26^ and Wang, *et al*.^56^ with some modification.

Danfeng-2 and Cabernet Sauvignon epicarp samples were used for protein extraction at each of the four developmental stages (GH, BV, V and R). The tissue was first finely ground and 1 g was then placed in a 10-ml tube containing 5 ml 10% m/v trichloroacetic acid in acetone, 5 mM DL-Dithiothreitol and 5 mM Phenylmethanesulfonyl fluoride, and the sample was held at −20 °C for 30 min. The sample was then centrifuged at 15,000 *g* at 4 °C for 30 min, and the pellet was washed three times in cold acetone and dried in a vacuum freeze drier (Labogene CS110-4). The dry pellet was then dissolved in extraction buffer^57^, thoroughly mixed by vortex and placed in a boiling water bath for 5 min and then in an ultra-sonicator (80 W) for 5 min^56^. A Bradford assay was used to determine the protein concentration^58^ and the quality of the protein samples was determined by SDS-PAGE gel electrophoresis^59^.

### Protein digestion and isobaric peptide labeling

For each sample, 100 μg of protein was denatured by adding half the corresponding volume of 2% SDS and reducing with an equal volume of 50 mM Tris-(2-carboxyethyl)-phosphine. Methyl methanethiosulfonate was used to block cysteine residues, as described in the iTRAQ Reagents kit (AB Sciex). For the key developmental stages of both *Vitis* species, sequencing-grade trypsin was used to digest the proteins at 37 °C for 16 h and the resulting peptides were labeled following the instructions for the iTRAQ reagent kit (Fig. 1). Three biological replicates were analyzed. After labeling, the eight samples were mixed.

### Peptide fractionation using strong cation exchange

Strong cation exchange (SCX) was used to fractionate the mixed labeled samples as previously described^46^. After separation, the 36 fractions collected were combined to yield 10 fractions. These were then vacuum-concentrated, re-suspended and desalted^56^.

### Nanoflow LC-MS/MS

Nanoflow LC-MS/MS were conducted as previously described by Zhong, *et al*.^60^ and Wang, *et al.* ^56^.

Each fraction described above was applied to a split-free, nanoflow LC system (EASY-nLC, Thermo scientific) with an EASY column (75 μm × 100 mm, 3 μm-C18, Thermo scientific) and eluted with a 120 min gradient (0–35% B in 100 min, 35–100% B in 8 min, 10% B in 12 min, where A is 0.1% formic acid dissolved in HPLC grade H_2_O and B is 84% acetonitrile (ACN), 0.1% formic acid in HPLC grade H_2_O at a flow rate of 250 nl/min) modified from Zhong^60^. The eluent was sprayed directly into a Q Exactive mass spectrometer (Thermo Scientific). MS data were acquired as previously described^56^.

### Sequence database retrieval

The MS/MS spectra were used to interrogate the proteins of *Vitis* in the Uniport database (http://www.uniprot.org/) (downloaded April 2014, 56,310 sequences) using MASCOT software (http://www.matrixscience.com). The MASCOT parameters were as previously described^56^. Peptide FDR ≤ 0.01 were generated for further analysis. We used Proteome Discoverer 1.4 (Thermo Scientific) for quantitative analysis with the following parameters: Protein Quantification: Use Only Unique Peptides, Experimental Bias: Normalize on Protein Median.

### Functional annotation

The STRAP editor (structural alignments of proteins) was used for annotation of the proteins identified^61^. Differently expressed proteins were defined as those with a 1.5-fold quantitation ratio (a quantitation ratio ≤ 0.67 is defined as down-regulated and a quantitation ratio ≥ 1.5 as up-regulated) with p value < 0.05. The following subsets of differentially expressed proteins analyzed were divided into subparts: Danfeng-2 GH vs Cabernet Sauvignon GH, Danfeng-2 BV vs Cabernet Sauvignon BV, Danfeng-2 V vs Cabernet Sauvignon V, and Danfeng-2 R vs Cabernet Sauvignon R. Venn diagrams of the data were drawn using VennPainter (https://github.com/linguoliang/VennPainter). Gene ontology analyses for differentially expressed proteins were retrieved from the Uniport database (http://www.uniprot.org/). Cytoscape was used for plotting the GO annotation of the differentially expressed proteins.

### Measurement of contents of resveratrol, piceid, anthocyanin and total flavonoid

The contents of resveratrol and piceid were determined using the method of Zhou, *et al*.^21^. Anthocyanin levels were measured as previously described^62^. The total flavonoid content was determined using ultraviolet spectroscopy^63^.

### RNA extraction and qRT-PCR

Total RNA was extracted using the method of Reid^64^ and the primer sets for each gene were designed using Primer Premier 5.0 (Primer, Canada) (Supplementary Table S1). qRT-PCR was carried out as Xu *et al*.^53^.

## Electronic supplementary material

Supplementary Figures and Tables

Supplementary Table s2

Supplementary Table s3

Supplementary Table s4
