# Supplementary material for: Resveratrol accumulation and its involvement in stilbene synthetic pathway of Chinese wild grapes during berry development using quantitative proteome analysis
Source: Sci Rep. 2017 Aug 24;7:9295. doi: 10.1038/s41598-017-10171-x (PMC5571159; doi:10.1038/s41598-017-10171-x)
Supplement: Supplementary file 1 — Supplementary Figures and Tables [file 41598_2017_10171_MOESM1_ESM.pdf]

Supplementary information

Resveratrol accumulation and its involvement in stilbene synthetic pathway of Chinese wild grapes during berry development using quantitative proteome analysis

Ruimin Li[a,b,c,†], Xiaoqing Xie[a,b,c,†], Fuli Ma[a,b,c,†], Dan Wang[a,b,c], Lan Wang[a,b,c], Jianxia Zhang [a,b,c], Yan Xu [a,b,c], Xiping Wang [a,b,c], Chaohong Zhang [a,b,c]*, Yuejin Wang[a,b,c]*

[a] College of Horticulture, Northwest A&F University, Yangling, Shaanxi, 712100, the People's Republic of China

[b] Key Laboratory of Horticultural Plant Biology and Germplasm Innovation in Northwest China, Ministry of Agriculture, Yangling, Shaanxi 712100, the People's Republic of China

[c] State Key Laboratory of Crop Stress Biology in Arid Areas, Northwest A&F University, Yangling, Shaanxi, 712100, the People's Republic of China

*Corresponding author: Chaohong Zhang

E-mail: zhangchaohong@nwsuaf.edu.cn

*Corresponding author: Yuejin Wang

E-mail: wangyj@nwsuaf.edu.cn

Tel: +86-29-87082522; Fax: +86-29-87082522

Supplementary Figures and Tables

Supplementary figure 1. Protein samples detected by SDS-PAGE gel. A1, A2, A3, three biological repeats of Green hard stage of *Vitis quinquangularis* accession Danfeng-2 and *V. vinifera* cv. Cabernet Sauvignon. B1, B2, B3, three biological repeats of Before Veraison stage of Danfeng-2. C1, C2, C3, three biological repeats of Veraison stage of Danfeng-2. D1, D2, D3, three biological repeats of Ripe stage of Danfeng-2. K1, K2, K3, three biological repeats of Green hard stage of Cabernet Sauvignon. L1, L2, L3, three biological repeats of Before Veraison stage of Cabernet Sauvignon. M1, M2, M3, three biological repeats of Veraison stage of Cabernet Sauvignon. N1, N2, N3, three biological repeats of Ripe stage of Cabernet Sauvignon.

Supplementary figure 2. Peptide fractionation using strong cation exchange. A,B,C represent three biological repeats of iTRAQ experiments.

Supplementary figure 3. Protein samples detected by Mass spectra (MALDI-TOF). A, Green hard stage of Danfeng-2. B, Before Veraison stage of Danfeng-2. C, Veraison stage of Danfeng-2. D, Ripe stage of Danfeng-2. E, Green hard stage of Cabernet Sauvignon. F, Before

Veraison stage of Cabernet Sauvignon. G, Veraison stage of Cabernet Sauvignon. H, Ripe stage of Cabernet Sauvignon.

Supplementary figure 4. Data repeatability of iTRAQ experiments of berry developmental stages of *Vitis quinquangularis* accession Danfeng-2 and *V. vinifera* cv. Cabernet Sauvignon. The Ratio at each development stage A) Green hard, B) Before veraison, C) Veraison, D) Ripe, of the three experiments between *Vitis quinquangularis* accession Danfeng-2 and *V. vinifera* cv. Cabernet Sauvignon are plotted in a correlation scatter diagram and the correlation coefficient R values were calculated. Three independent biological replicates are labeled as a, b, and c. 113, 114, 115, 116, 117, 118, 119, 121, samples number of different developmental stages of *Vitis quinquangularis* accession Danfeng-2 and *V. vinifera* cv. Cabernet Sauvignon (as indicated in Fig.1A).

Supplementary Table S1. Primers used in the present study.

Note: Primers were designed with Primer Premier 5.0 and used for qRT-PCR in Figure. 8 and Supplementary Table S6.

Supplementary Table S2. List of peptides identified and quantified in the iTRAQ experiments.

Note: Three independent biological replicates were labeled as a, b, and c. 113, 114, 115, 116, 117, 118, 119, 121, samples number of different developmental stages of Danfeng-2 and Cabernet Sauvignon (as indicated in Fig.1A).

Supplementary Table S3. List of proteins identified and quantified in the iTRAQ experiments.

Note: Three independent biological replicates were labeled as a, b, and c. 113, 114, 115, 116, 117, 118, 119, 121, samples number of different developmental stages of Danfeng-2 and Cabernet Sauvignon (as indicated in Fig.1A).

Supplementary Table S4. Differentially expressed proteins between Danfeng-2 and Cabernet Sauvignon at four developmental stages.

Note: *, significant differentially expressed proteins (p-value < 0.05). ‘up’ means up-regulated profile and ‘down’ means down-regulated profile in Danfeng-2. GH, green hard, BV, before veraison, V, veraison, and R, ripe. Three independent biological replicates were labeled as a, b, and c. 113, 114, 115, 116, 117, 118, 119, 121, samples number of different developmental stages of Danfeng-2 and Cabernet Sauvignon (as indicated in Fig.1A).

Supplementary Table S5 Enriched GO terms of differential expressed proteins between Danfeng-2 and Cabernet Sauvignon.

Note: Enriched GO terms were generated in agriGO with customized annotation as reference. *p-values* were converted with *Z*-score generated by *z*-test. FDR were generated as p-value with multiple test correction.

Supplementary Table S6. Comparison of protein and mRNA expression pattern of 15 candidate proteins (genes) at different developmental stages.

Note: RT-PCR data are log2 normalized $2^{-\Delta\Delta Ct}$ values. Relationships of proteins with qRT-PCR transcripts are indicated.

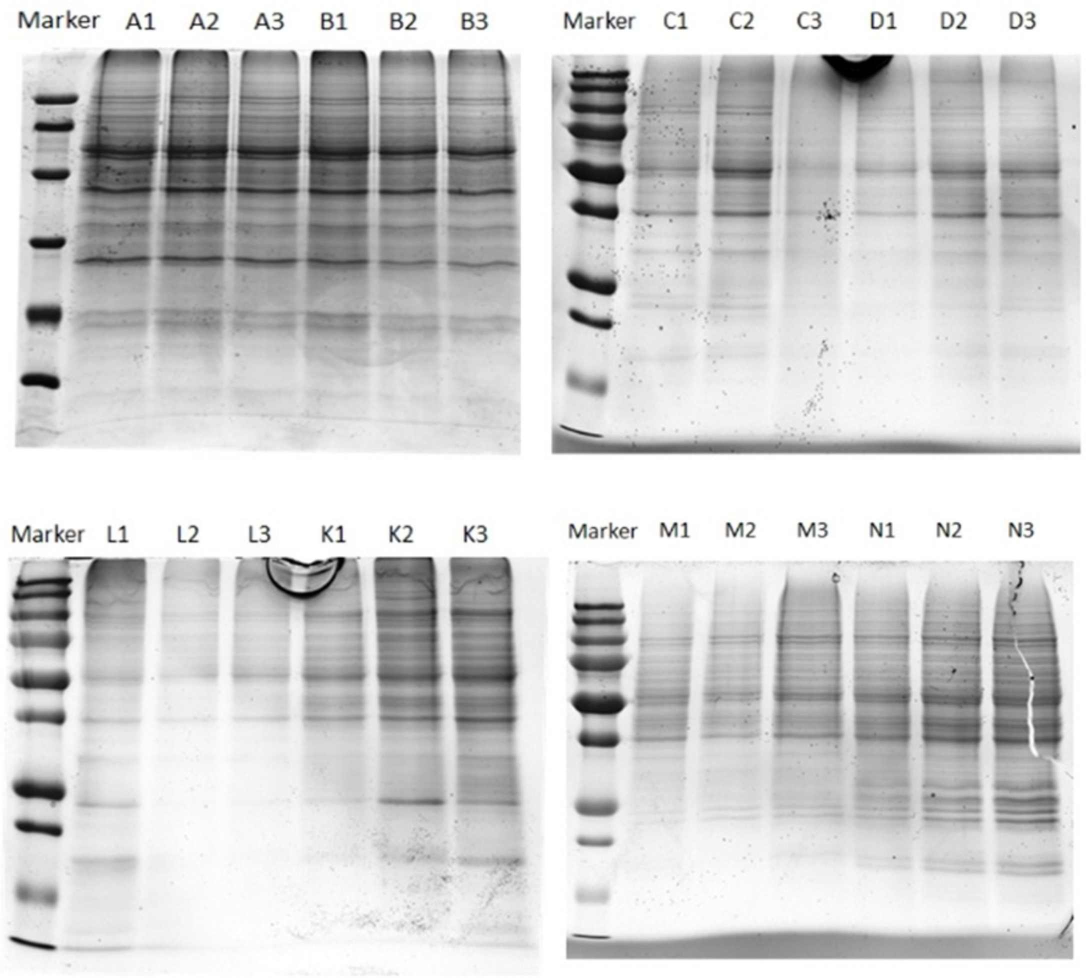

Supplementary figure 1. Protein samples detected by SDS-PAGE gel. A1, A2, A3, three biological repeats of Green hard stage of *Vitis*

quinquangularis accession Danfeng-2 and *V. vinifera* cv. Cabernet Sauvignon. B1, B2, B3, three biological repeats of Before Veraison stage of Danfeng-2. C1, C2, C3, three biological repeats of Veraison stage of Danfeng-2. D1, D2, D3, three biological repeats of Ripe stage of Danfeng-2. K1, K2, K3, three biological repeats of Green hard stage of Cabernet Sauvignon. L1, L2, L3, three biological repeats of Before Veraison stage of Cabernet Sauvignon. M1, M2, M3, three biological repeats of Veraison stage of Cabernet Sauvignon. N1, N2, N3, three biological repeats of Ripe stage of Cabernet Sauvignon.

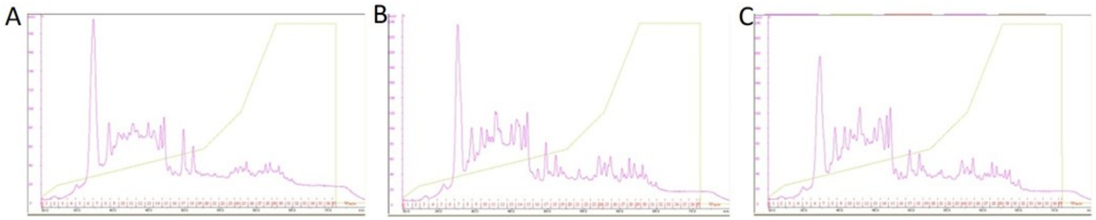

Supplementary figure 2. Peptide fractionation using strong cation exchange. A,B,C represent three biological repeats of iTRAQ experiments.

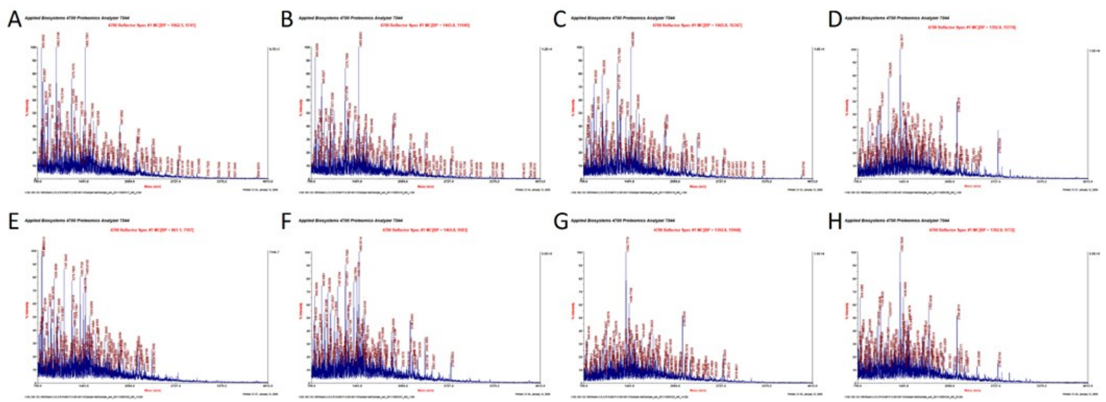

Supplementary figure 3. Protein samples detected by Mass spectra (MALDI-TOF). A, Green hard stage of Danfeng-2. B, Before Veraison stage of Danfeng-2. C, Veraison stage of Danfeng-2. D, Ripe stage of Danfeng-2. E, Green hard stage of Cabernet Sauvignon. F, Before Veraison stage of Cabernet Sauvignon. G, Veraison stage of Cabernet Sauvignon. H, Ripe stage of Cabernet Sauvignon.

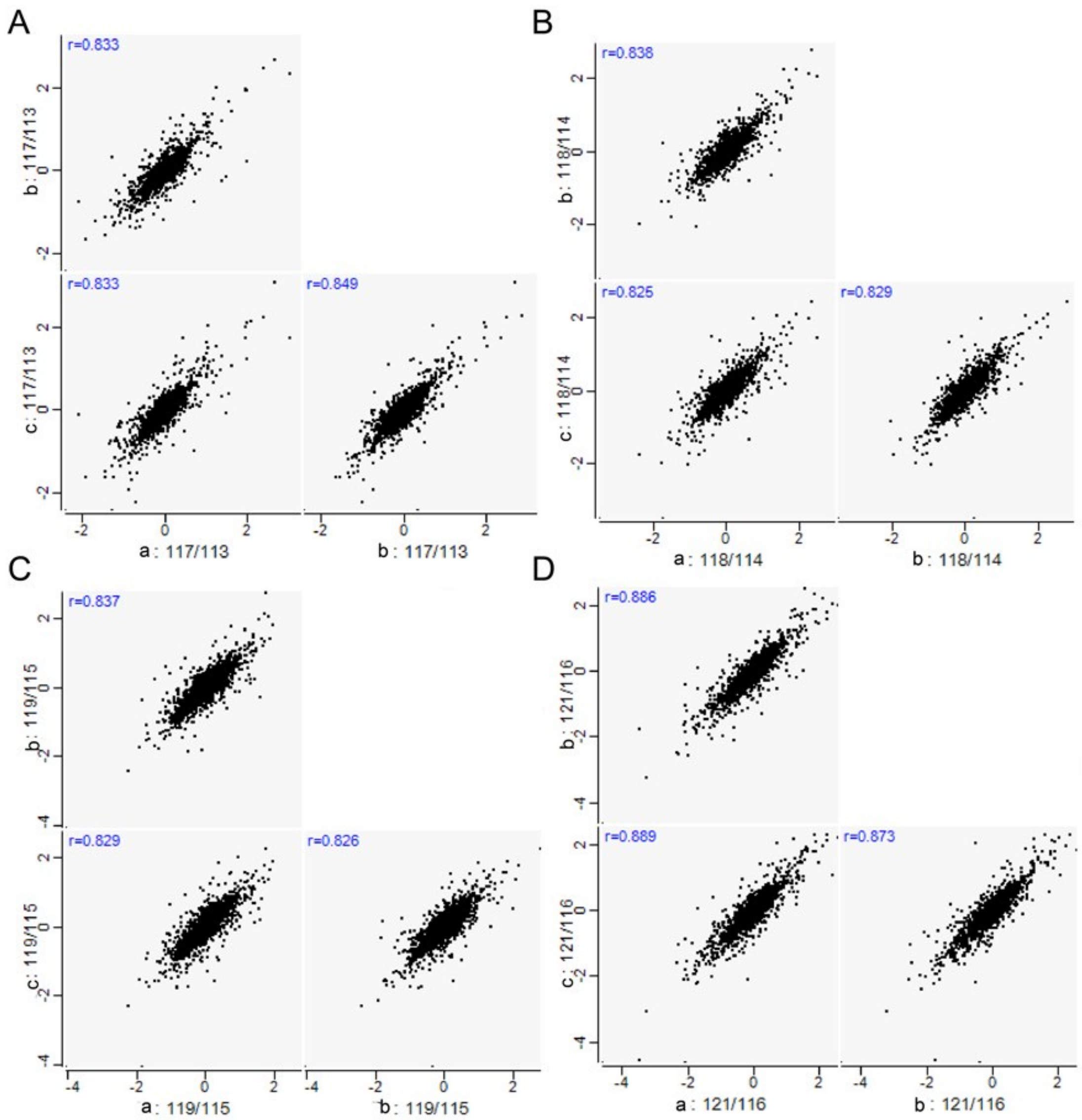
A
r=0.833
b: 117/113
r=0.833
r=0.849
c: 117/113
a : 117/113
b : 117/113
B
r=0.838
b: 118/114
r=0.825
r=0.829
c: 118/114
a : 118/114
b : 118/114
C
r=0.837
b: 119/115
r=0.829
r=0.826
c: 119/115
a : 119/115
b : 119/115
D
r=0.886
b: 121/116
r=0.889
r=0.873
c: 121/116
a : 121/116
b : 121/116

Supplementary figure 4. Data repeatability of iTRAQ experiments of berry developmental stages of *Vitis quinquangularis* accession Danfeng-2 and *V. vinifera* cv. Cabernet Sauvignon. The Ratio at each development stage A) Green hard, B) Before veraison, C) Veraison, D) Ripe, of the three experiments between *Vitis quinquangularis* accession Danfeng-2 and *V. vinifera* cv. Cabernet Sauvignon are plotted in a correlation scatter diagram and the correlation coefficient R values were calculated. Three independent biological replicates are labeled as a, b, and c. 113, 114, 115, 116, 117, 118, 119, 121, samples number of different developmental stages of *Vitis quinquangularis* accession Danfeng-2 and *V. vinifera* cv. Cabernet Sauvignon (as indicated in Fig.1A).

Supplementary Table s1. Primers used in the present study

Gene Name_Forward or _Reverse Primer	Primer Sequence
UFGT_Forward	TAACACATTGTGGATGGAACTCAT
UFGT_Reverse	ACCTTCAATTCTCACTCCAATCTC
MYBA1_Forward	GAGGGTGATTTTCCATTTGAT
MYBA1_Reverse	CAAGAACAACTTTTGAACTTAAACAT
MYB14_Forward	TCTGAGGCCGGATATCAAAC
MYB14_Reverse	GGGACGCATCAAGAGAGTGT
MYB15_Forward	CAAGAATGAACAGATGGAGGAG
MYB15_Reverse	TCTGCGACTGCTGGGAAA
MYB5a_Forward	CTAGAACTGTCTGGGAACCT
MYB5a_Reverse	TGCAAGGATCCATTTCACATAC
MYB5b_Forward	TGACAGCCGGTGTTCTTTAAT
MYB5b_Reverse	AGCATACTAACACAACAACACAACC
MYBPA1_Forward	AGATCAACTGGTTATGCTTGCT
MYBPA1_Reverse	AACACAAATGTACATCGCACAC
CHS_Forward	CATCACAAATAGCGAACACAAG
CHS_Reverse	CCTAGCATCCAGGGAAGC
STS_Forward	CAAGCCCTTTTTGGTGATG
STS_Reverse	CCACAAGTGAAAGGTGAGTCC
F35H1_Forward	TTCTTGAAAATGGGCACTAACA
F35H1_Reverse	CGCTCGCTGGCACAACT
F35H2_Forward	TGCTGATGGAACGCCTG
F35H2_Reverse	CATTTGTCTGACTTTTTTGGG
F3H_Forward	TGTCTGGTGGCAAGAAAGG
F3H_Reverse	CAAGCATTGGTCAAAGCGT

LDOX_Forward	TGGAGTGGGGTGTGATGC
LDOX_Reverse	GGTGTCTTAGGCCAGATGGT
CHI1_Forward	GGAAGGGTAAATCTGGAAAGG
CHI1_Reverse	GCATGAAGTTGCAGGAAAATG
CHI2_Forward	GCTCCACCAACGACCTCTT
CHI2_Reverse	TCCTCCACAGTCTTGCCCT
DFR_Forward	GCGGAGACGCATCTGACT
DFR_Reverse	GCCCAACATCCCTTCAATA
C4H_Forward	ACCACCTGAACCTCTCCGA
C4H_Reverse	CGCCAATGCTCACCGTA
UBC32_Forward	CAAGAGTGGCTGACAGGATAG
UBC32_Reverse	CTTGATACAGGGCTGGGATTAG
LRR_Forward	GGCCAACCTGGTAAACAGAATA
LRR_Reverse	CGGAGGTTCTGCAGGTAAAT
GRP_Forward	CTGTTGTTCTCATCCTGTCCTC
GRP_Reverse	CCCATCGTGCTTCTCATCTT
PIP2;3_Forward	CTGGAGTGACCATTGGGTATTT
PIP2;3_Reverse	AACAGCTCCAGCTCTCAATATG
USP_Forward	GTTCTTGGGAGCAGAGGTTTAG
USP_Reverse	TCACAACTGTAACTGGACATGAG
RSGT_Forward	CTACCATGGCTTAGTCCCTTTC
RSGT_Reverse	AGCTGGCGATTTCATCATACT
GAPDH_Forward	TTCTCGTTGAGGGCTATTCCA
GAPDH_Reverse	CCACAGACTTCATCGGTGACA

Note: Primers were designed with Primer Premier 5.0 and used for qRT-PCR in Figure. 8 and Supplementary Table S6.

Supplementary Table s5. Enriched GO terms of differential expressed proteins between Danfeng-2 and Cabernet Sauvignon

GO term	Ontology	Description	Number in input list	Number in BG/Ref	p-value	FDR
GO:0015979	P	photosynthesis	40	162	6.30E-35	1.50E-31
GO:0044237	P	cellular metabolic process	239	8722	2.90E-32	3.50E-29
GO:0009987	P	cellular process	283	11684	5.10E-31	4.10E-28
GO:0008152	P	metabolic process	266	10614	1.00E-30	6.10E-28
GO:0044262	P	cellular carbohydrate metabolic process	49	417	3.60E-29	1.80E-26
GO:0006082	P	organic acid metabolic process	62	860	7.40E-26	3.00E-23
GO:0034641	P	cellular nitrogen compound metabolic process	49	506	1.00E-25	3.50E-23
GO:0043436	P	oxoacid metabolic process	61	859	3.90E-25	1.10E-22
GO:0019752	P	carboxylic acid metabolic process	61	859	3.90E-25	1.10E-22
GO:0005975	P	carbohydrate metabolic process	61	866	5.90E-25	1.40E-22
GO:0042180	P	cellular ketone metabolic process	61	882	1.40E-24	3.20E-22
GO:000	P	alcohol metabolic process	37	270	2.30E	4.40E

6066					-24	-22
GO:000 6519	P	cellular amino acid and derivative metabolic process	54	682	2.20E -24	4.40E -22
GO:000 5996	P	monosaccharide metabolic process	30	168	6.70E -23	1.20E -20
GO:004 4238	P	primary metabolic process	221	8995	1.40E -22	2.30E -20
GO:000 6091	P	generation of precursor metabolites and energy	35	285	1.10E -21	1.60E -19
GO:004 4248	P	cellular catabolic process	52	746	3.40E -21	4.90E -19
GO:003 4622	P	cellular macromolecular complex assembly	28	200	6.00E -19	8.10E -17
GO:001 9684	P	photosynthesis, light reaction	22	103	1.40E -18	1.70E -16
GO:006 5003	P	macromolecular complex assembly	28	210	1.90E -18	2.30E -16
GO:004 4106	P	cellular amine metabolic process	37	438	7.30E -18	8.40E -16
GO:003 4621	P	cellular macromolecular complex subunit organization	28	224	8.80E -18	9.70E -16
GO:004 4085	P	cellular component biogenesis	41	571	2.40E -17	2.60E -15
GO:004 3933	P	macromolecular complex subunit organization	28	235	2.70E -17	2.80E -15
GO:000	P	amine metabolic process	39	521	4.10E	4.00E

9308					-17	-15
GO:0009069	P	serine family amino acid metabolic process	17	54	4.60E-17	4.30E-15
GO:0006790	P	sulfur metabolic process	27	220	5.20E-17	4.70E-15
GO:0019318	P	hexose metabolic process	22	126	5.80E-17	5.10E-15
GO:0006520	P	cellular amino acid metabolic process	35	430	1.70E-16	1.40E-14
GO:0022607	P	cellular component assembly	28	265	4.50E-16	3.70E-14
GO:0006996	P	organelle organization	41	640	9.80E-16	7.70E-14
GO:0009056	P	catabolic process	59	1307	1.70E-15	1.30E-13
GO:0006006	P	glucose metabolic process	18	86	2.50E-15	1.90E-13
GO:0046394	P	carboxylic acid biosynthetic process	32	417	1.60E-14	1.10E-12
GO:0016053	P	organic acid biosynthetic process	32	417	1.60E-14	1.10E-12
GO:0006090	P	pyruvate metabolic process	12	25	3.40E-14	2.30E-12
GO:0016052	P	carbohydrate catabolic process	19	128	1.10E-13	7.10E-12
GO:000	P	cellular amino acid derivative metabolic process	27	315	1.60E	1.00E

6575					-13	-11
GO:000 0096	P	sulfur amino acid metabolic process	16	84	3.20E -13	2.00E -11
GO:002 2613	P	ribonucleoprotein complex biogenesis	24	253	5.00E -13	3.00E -11
GO:002 2618	P	ribonucleoprotein complex assembly	10	16	6.40E -13	3.80E -11
GO:004 4271	P	cellular nitrogen compound biosynthetic process	29	394	7.20E -13	4.20E -11
GO:000 0097	P	sulfur amino acid biosynthetic process	13	49	1.50E -12	8.20E -11
GO:004 4272	P	sulfur compound biosynthetic process	17	115	2.30E -12	1.30E -10
GO:000 8652	P	cellular amino acid biosynthetic process	21	202	2.90E -12	1.60E -10
GO:000 9070	P	serine family amino acid biosynthetic process	11	29	3.10E -12	1.60E -10
GO:000 9309	P	amine biosynthetic process	22	229	3.70E -12	1.90E -10
GO:000 9058	P	biosynthetic process	127	5118	3.90E -12	2.00E -10
GO:001 9748	P	secondary metabolic process	31	489	4.40E -12	2.20E -10
GO:000 6725	P	cellular aromatic compound metabolic process	28	399	5.20E -12	2.50E -10
GO:004	P	cellular carbohydrate catabolic process	17	125	7.50E	3.60E

4275					-12	-10
GO:0032787	P	monocarboxylic acid metabolic process	28	408	8.50E-12	4.00E-10
GO:0016043	P	cellular component organization	49	1179	8.70E-12	4.00E-10
GO:0019344	P	cysteine biosynthetic process	10	24	1.40E-11	6.30E-10
GO:0055086	P	nucleobase, nucleoside and nucleotide metabolic process	21	221	1.40E-11	6.30E-10
GO:0006534	P	cysteine metabolic process	10	24	1.40E-11	6.30E-10
GO:0015977	P	carbon fixation	9	16	1.90E-11	8.20E-10
GO:0051186	P	cofactor metabolic process	24	308	2.30E-11	9.90E-10
GO:0046483	P	heterocycle metabolic process	29	460	2.50E-11	1.00E-09
GO:0006081	P	cellular aldehyde metabolic process	10	27	3.70E-11	1.50E-09
GO:0006007	P	glucose catabolic process	14	83	4.20E-11	1.70E-09
GO:0019320	P	hexose catabolic process	14	84	4.80E-11	1.90E-09
GO:0046365	P	monosaccharide catabolic process	14	84	4.80E-11	1.90E-09
GO:003	P	cellular response to oxidative stress	8	11	5.80E	2.20E

4599					-11	-09
GO:004 2254	P	ribosome biogenesis	21	241	6.20E -11	2.30E -09
GO:004 6164	P	alcohol catabolic process	14	89	9.60E -11	3.50E -09
GO:004 4265	P	cellular macromolecule catabolic process	28	465	1.50E -10	5.40E -09
GO:001 9288	P	isopentenyl diphosphate biosynthetic process, mevalonate-independent pathway	8	13	1.50E -10	5.40E -09
GO:001 9682	P	glyceraldehyde-3-phosphate metabolic process	8	13	1.50E -10	5.40E -09
GO:000 8610	P	lipid biosynthetic process	27	439	2.00E -10	7.10E -09
GO:005 1179	P	localization	62	1922	3.40E -10	1.20E -08
GO:005 1649	P	establishment of localization in cell	29	525	4.60E -10	1.60E -08
GO:005 1234	P	establishment of localization	60	1851	5.70E -10	1.90E -08
GO:005 1641	P	cellular localization	30	569	6.60E -10	2.20E -08
GO:000 9813	P	flavonoid biosynthetic process	12	69	7.60E -10	2.40E -08
GO:004 6490	P	isopentenyl diphosphate metabolic process	8	17	7.70E -10	2.40E -08
GO:000	P	isopentenyl diphosphate biosynthetic process	8	17	7.70E	2.40E

9240					-10	-08
GO:0044249	P	cellular biosynthetic process	116	4925	1.00E-09	3.20E-08
GO:0034637	P	cellular carbohydrate biosynthetic process	17	177	1.10E-09	3.30E-08
GO:0006732	P	coenzyme metabolic process	17	188	2.50E-09	7.50E-08
GO:0009812	P	flavonoid metabolic process	12	78	2.60E-09	7.90E-08
GO:0016051	P	carbohydrate biosynthetic process	20	277	3.70E-09	1.10E-07
GO:0044255	P	cellular lipid metabolic process	30	618	4.00E-09	1.20E-07
GO:0044267	P	cellular protein metabolic process	89	3487	4.00E-09	1.20E-07
GO:0019725	P	cellular homeostasis	16	174	5.90E-09	1.70E-07
GO:0044264	P	cellular polysaccharide metabolic process	14	127	6.40E-09	1.80E-07
GO:0005976	P	polysaccharide metabolic process	15	152	7.30E-09	2.00E-07
GO:0006753	P	nucleoside phosphate metabolic process	16	180	9.20E-09	2.50E-07
GO:0009117	P	nucleotide metabolic process	16	180	9.20E-09	2.50E-07
GO:004	P	glucan metabolic process	12	89	1.00E	2.70E

4042					-08	-07
GO:0016072	P	rRNA metabolic process	13	110	1.00E-08	2.70E-07
GO:0006364	P	rRNA processing	13	110	1.00E-08	2.70E-07
GO:0006950	P	response to stress	66	2320	1.20E-08	3.00E-07
GO:0042592	P	homeostatic process	17	216	1.70E-08	4.40E-07
GO:0006457	P	protein folding	19	275	1.80E-08	4.50E-07
GO:0006810	P	transport	56	1846	2.10E-08	5.30E-07
GO:0006629	P	lipid metabolic process	34	841	2.90E-08	7.30E-07
GO:0046165	P	alcohol biosynthetic process	9	44	3.00E-08	7.40E-07
GO:0006749	P	glutathione metabolic process	6	10	3.70E-08	8.90E-07
GO:0034614	P	cellular response to reactive oxygen species	6	10	3.70E-08	8.90E-07
GO:0043623	P	cellular protein complex assembly	13	124	3.80E-08	9.20E-07
GO:0033036	P	macromolecule localization	24	462	4.50E-08	1.10E-06
GO:001	P	protein metabolic process	94	4009	7.50E	1.80E

9538					-08	-06
GO:0006073	P	cellular glucan metabolic process	11	87	7.60E-08	1.80E-06
GO:0009886	P	post-embryonic morphogenesis	8	35	8.40E-08	1.90E-06
GO:0006461	P	protein complex assembly	13	134	8.80E-08	2.00E-06
GO:0042440	P	pigment metabolic process	13	134	8.80E-08	2.00E-06
GO:0070271	P	protein complex biogenesis	13	134	8.80E-08	2.00E-06
GO:0008299	P	isoprenoid biosynthetic process	13	135	9.50E-08	2.10E-06
GO:0046148	P	pigment biosynthetic process	12	112	1.00E-07	2.30E-06
GO:0010027	P	thylakoid membrane organization	7	23	1.00E-07	2.30E-06
GO:0009668	P	plastid membrane organization	7	23	1.00E-07	2.30E-06
GO:0009765	P	photosynthesis, light harvesting	7	26	2.10E-07	4.60E-06
GO:0042398	P	cellular amino acid derivative biosynthetic process	16	233	2.60E-07	5.40E-06
GO:0042743	P	hydrogen peroxide metabolic process	7	27	2.60E-07	5.60E-06
GO:001	P	aromatic compound biosynthetic process	16	237	3.20E	6.60E

9438					-07	-06
GO:0006605	P	protein targeting	13	155	4.20E-07	8.70E-06
GO:0046907	P	intracellular transport	22	463	6.80E-07	1.40E-05
GO:0006518	P	peptide metabolic process	7	32	7.10E-07	1.50E-05
GO:0008654	P	phospholipid biosynthetic process	10	88	7.30E-07	1.50E-05
GO:0006720	P	isoprenoid metabolic process	13	168	9.80E-07	2.00E-05
GO:0009699	P	phenylpropanoid biosynthetic process	12	141	1.00E-06	2.00E-05
GO:0031399	P	regulation of protein modification process	5	10	1.10E-06	2.10E-05
GO:0070301	P	cellular response to hydrogen peroxide	5	10	1.10E-06	2.10E-05
GO:0010207	P	photosystem II assembly	5	10	1.10E-06	2.10E-05
GO:0009607	P	response to biotic stimulus	26	638	1.10E-06	2.10E-05
GO:0034470	P	ncRNA processing	13	170	1.10E-06	2.10E-05
GO:0009657	P	plastid organization	11	119	1.30E-06	2.60E-05
GO:000	P	protein localization	20	408	1.40E	2.60E

8104					-06	-05
GO:0006544	P	glycine metabolic process	6	22	1.50E-06	2.80E-05
GO:0006644	P	phospholipid metabolic process	11	121	1.60E-06	2.90E-05
GO:0009250	P	glucan biosynthetic process	8	54	1.60E-06	2.90E-05
GO:0046395	P	carboxylic acid catabolic process	10	98	1.80E-06	3.20E-05
GO:0016054	P	organic acid catabolic process	10	98	1.80E-06	3.20E-05
GO:0045184	P	establishment of protein localization	19	381	2.00E-06	3.60E-05
GO:0015031	P	protein transport	19	381	2.00E-06	3.60E-05
GO:0009057	P	macromolecule catabolic process	33	982	2.50E-06	4.40E-05
GO:0019637	P	organophosphate metabolic process	11	130	3.00E-06	5.30E-05
GO:0051789	P	response to protein stimulus	5	14	3.90E-06	6.80E-05
GO:0006098	P	pentose-phosphate shunt	6	28	5.00E-06	8.70E-05
GO:0010498	P	proteasomal protein catabolic process	5	15	5.20E-06	8.90E-05
GO:004	P	cellular amide metabolic process	7	45	5.40E	9.20E

3603					-06	-05
GO:0006740	P	NADPH regeneration	6	29	6.00E-06	0.0001
GO:0006800	P	oxygen and reactive oxygen species metabolic process	8	66	6.10E-06	0.0001
GO:0009698	P	phenylpropanoid metabolic process	12	175	8.10E-06	0.00014
GO:0006886	P	intracellular protein transport	16	311	8.80E-06	0.00015
GO:0009310	P	amine catabolic process	7	49	8.90E-06	0.00015
GO:0044260	P	cellular macromolecule metabolic process	125	6447	9.10E-06	0.00015
GO:0006739	P	NADP metabolic process	6	32	9.90E-06	0.00016
GO:0045454	P	cell redox homeostasis	8	72	1.10E-05	0.00018
GO:0034613	P	cellular protein localization	16	322	1.30E-05	0.00021
GO:0000272	P	polysaccharide catabolic process	5	19	1.40E-05	0.00022
GO:0019252	P	starch biosynthetic process	5	19	1.40E-05	0.00022
GO:0033554	P	cellular response to stress	18	399	1.40E-05	0.00022
GO:004	P	response to hydrogen peroxide	7	53	1.40E	0.000

2542					-05	22
GO:000<br>9404	P	toxin metabolic process	7	53	1.40E<br>-05	0.000<br>22
GO:000<br>9407	P	toxin catabolic process	7	53	1.40E<br>-05	0.000<br>22
GO:000<br>6631	P	fatty acid metabolic process	13	225	1.90E<br>-05	0.000<br>3
GO:007<br>0838	P	divalent metal ion transport	5	21	2.10E<br>-05	0.000<br>31
GO:005<br>1640	P	organelle localization	5	21	2.10E<br>-05	0.000<br>31
GO:004<br>6700	P	heterocycle catabolic process	6	38	2.40E<br>-05	0.000<br>36
GO:000<br>6733	P	oxidoreduction coenzyme metabolic process	7	58	2.40E<br>-05	0.000<br>36
GO:004<br>5893	P	positive regulation of transcription, DNA-dependent	5	22	2.50E<br>-05	0.000<br>37
GO:007<br>0727	P	cellular macromolecule localization	16	341	2.60E<br>-05	0.000<br>38
GO:000<br>6807	P	nitrogen compound metabolic process	81	3826	3.00E<br>-05	0.000<br>44
GO:005<br>1254	P	positive regulation of RNA metabolic process	5	23	3.00E<br>-05	0.000<br>44
GO:003<br>4976	P	response to endoplasmic reticulum stress	5	23	3.00E<br>-05	0.000<br>44
GO:003	P	cellular cation homeostasis	8	84	3.10E	0.000

0003					-05	44
GO:000<br>0302	P	response to reactive oxygen species	8	85	3.30E<br>-05	0.000<br>48
GO:001<br>9321	P	pentose metabolic process	6	41	3.50E<br>-05	0.000<br>49
GO:004<br>6496	P	nicotinamide nucleotide metabolic process	6	41	3.50E<br>-05	0.000<br>49
GO:000<br>5982	P	starch metabolic process	6	41	3.50E<br>-05	0.000<br>49
GO:000<br>6769	P	nicotinamide metabolic process	6	41	3.50E<br>-05	0.000<br>49
GO:000<br>6576	P	cellular biogenic amine metabolic process	7	62	3.60E<br>-05	0.000<br>5
GO:000<br>9064	P	glutamine family amino acid metabolic process	6	42	3.90E<br>-05	0.000<br>54
GO:001<br>6044	P	cellular membrane organization	9	114	3.90E<br>-05	0.000<br>54
GO:002<br>2900	P	electron transport chain	8	88	4.20E<br>-05	0.000<br>57
GO:005<br>5080	P	cation homeostasis	8	90	4.80E<br>-05	0.000<br>66
GO:004<br>4036	P	cell wall macromolecule metabolic process	6	45	5.60E<br>-05	0.000<br>76
GO:003<br>3692	P	cellular polysaccharide biosynthetic process	8	92	5.60E<br>-05	0.000<br>76
GO:000	P	aromatic amino acid family metabolic process	7	68	6.10E	0.000

9072					-05	82
GO:001<br>9362	P	pyridine nucleotide metabolic process	6	46	6.20E<br>-05	0.000<br>83
GO:000<br>6464	P	protein modification process	39	1474	6.40E<br>-05	0.000<br>84
GO:003<br>4660	P	ncRNA metabolic process	13	254	6.30E<br>-05	0.000<br>84
GO:000<br>9165	P	nucleotide biosynthetic process	9	122	6.40E<br>-05	0.000<br>85
GO:000<br>9063	P	cellular amino acid catabolic process	6	47	7.00E<br>-05	0.000<br>9
GO:003<br>2268	P	regulation of cellular protein metabolic process	6	47	7.00E<br>-05	0.000<br>9
GO:000<br>6873	P	cellular ion homeostasis	8	96	7.40E<br>-05	0.000<br>96
GO:005<br>5082	P	cellular chemical homeostasis	8	97	7.90E<br>-05	0.001
GO:005<br>1188	P	cofactor biosynthetic process	11	191	8.60E<br>-05	0.001<br>1
GO:000<br>9116	P	nucleoside metabolic process	6	50	9.50E<br>-05	0.001<br>2
GO:004<br>3170	P	macromolecule metabolic process	130	7127	0.000<br>1	0.001<br>3
GO:000<br>9891	P	positive regulation of biosynthetic process	7	76	0.000<br>12	0.001<br>4
GO:000	P	oligosaccharide metabolic process	6	52	0.000	0.001

9311					12	4
GO:0019220	P	regulation of phosphate metabolic process	6	52	0.00012	0.0014
GO:0051174	P	regulation of phosphorus metabolic process	6	52	0.00012	0.0014
GO:0050801	P	ion homeostasis	8	103	0.00012	0.0014
GO:0031328	P	positive regulation of cellular biosynthetic process	7	76	0.00012	0.0014
GO:0031408	P	oxylipin biosynthetic process	5	32	0.00012	0.0015
GO:0051246	P	regulation of protein metabolic process	6	54	0.00014	0.0017
GO:0055114	P	oxidation reduction	11	203	0.00014	0.0017
GO:0065008	P	regulation of biological quality	22	665	0.00014	0.0017
GO:0006952	P	defense response	24	766	0.00016	0.0019
GO:0006633	P	fatty acid biosynthetic process	9	140	0.00017	0.0021
GO:0009119	P	ribonucleoside metabolic process	5	35	0.00018	0.0021
GO:0050790	P	regulation of catalytic activity	9	141	0.00018	0.0021
GO:000	P	glycolysis	6	57	0.000	0.002

6096					18	2
GO:0009820	P	alkaloid metabolic process	6	57	0.00018	0.0022
GO:0000271	P	polysaccharide biosynthetic process	8	112	0.0002	0.0023
GO:0006396	P	RNA processing	17	453	0.0002	0.0023
GO:0015992	P	proton transport	6	59	0.00022	0.0025
GO:0006818	P	hydrogen transport	6	59	0.00022	0.0025
GO:0034220	P	ion transmembrane transport	6	59	0.00022	0.0025
GO:0016042	P	lipid catabolic process	7	85	0.00022	0.0025
GO:0006979	P	response to oxidative stress	14	332	0.00024	0.0027
GO:0009206	P	purine ribonucleoside triphosphate biosynthetic process	6	60	0.00024	0.0027
GO:0009201	P	ribonucleoside triphosphate biosynthetic process	6	60	0.00024	0.0027
GO:0051716	P	cellular response to stimulus	25	840	0.00025	0.0028
GO:0009199	P	ribonucleoside triphosphate metabolic process	6	61	0.00026	0.0029
GO:000	P	purine ribonucleoside triphosphate metabolic process	6	61	0.000	0.002

9205					26	9
GO:003 1407	P	oxylipin metabolic process	5	39	0.000 28	0.003 1
GO:000 9145	P	purine nucleoside triphosphate biosynthetic process	6	62	0.000 28	0.003 1
GO:000 9260	P	ribonucleotide biosynthetic process	7	89	0.000 29	0.003 2
GO:000 9142	P	nucleoside triphosphate biosynthetic process	6	63	0.000 3	0.003 3
GO:000 9144	P	purine nucleoside triphosphate metabolic process	6	63	0.000 3	0.003 3
GO:000 6164	P	purine nucleotide biosynthetic process	7	90	0.000 31	0.003 3
GO:000 6811	P	ion transport	16	427	0.000 31	0.003 3
GO:006 5009	P	regulation of molecular function	9	153	0.000 32	0.003 4
GO:003 1325	P	positive regulation of cellular metabolic process	7	91	0.000 33	0.003 5
GO:000 9893	P	positive regulation of metabolic process	7	92	0.000 35	0.003 7
GO:000 6163	P	purine nucleotide metabolic process	7	92	0.000 35	0.003 7
GO:000 9141	P	nucleoside triphosphate metabolic process	6	65	0.000 36	0.003 7
GO:001	P	heterocycle biosynthetic process	8	123	0.000	0.003

8130					36	8
GO:001 5674	P	di-, tri-valent inorganic cation transport	5	43	0.000 42	0.004 4
GO:001 6192	P	vesicle-mediated transport	12	272	0.000 44	0.004 6
GO:000 9259	P	ribonucleotide metabolic process	7	96	0.000 44	0.004 6
GO:004 3412	P	macromolecule modification	39	1636	0.000 49	0.005
GO:000 8643	P	carbohydrate transport	5	45	0.000 51	0.005 2
GO:000 9767	P	photosynthetic electron transport chain	5	46	0.000 56	0.005 7
GO:000 7264	P	small GTPase mediated signal transduction	5	46	0.000 56	0.005 7
GO:004 3094	P	cellular metabolic compound salvage	6	73	0.000 63	0.006 3
GO:000 9658	P	chloroplast organization	6	73	0.000 63	0.006 3
GO:004 8878	P	chemical homeostasis	8	136	0.000 68	0.006 8
GO:001 6137	P	glycoside metabolic process	7	104	0.000 7	0.006 9
GO:005 0896	P	response to stimulus	78	4057	0.000 8	0.007 9
GO:000	P	chromatin organization	9	175	0.000	0.008

6325					81	
GO:0009152	P	purine ribonucleotide biosynthetic process	6	77	0.00082	0.0081
GO:0051276	P	chromosome organization	10	216	0.00091	0.0089
GO:0043687	P	post-translational protein modification	31	1248	0.00097	0.0095
GO:0009150	P	purine ribonucleotide metabolic process	6	80	0.00099	0.0096
GO:0006333	P	chromatin assembly or disassembly	6	82	0.0011	0.011
GO:0015672	P	monovalent inorganic cation transport	7	115	0.0012	0.012
GO:0006334	P	nucleosome assembly	5	56	0.0013	0.012
GO:0034728	P	nucleosome organization	5	56	0.0013	0.012
GO:0017038	P	protein import	6	86	0.0014	0.013
GO:0006754	P	ATP biosynthetic process	5	58	0.0015	0.014
GO:0046034	P	ATP metabolic process	5	59	0.0016	0.015
GO:0006778	P	porphyrin metabolic process	6	89	0.0017	0.016
GO:003	P	chromatin assembly	5	60	0.001	0.016

1497					7	
GO:006<br>5004	P	protein-DNA complex assembly	5	60	0.001<br>7	0.016
GO:001<br>0035	P	response to inorganic substance	11	279	0.001<br>8	0.017
GO:003<br>3013	P	tetrapyrrole metabolic process	6	93	0.002	0.019
GO:000<br>9314	P	response to radiation	18	613	0.002<br>1	0.019
GO:001<br>0628	P	positive regulation of gene expression	5	63	0.002<br>1	0.019
GO:004<br>5941	P	positive regulation of transcription	5	63	0.002<br>1	0.019
GO:005<br>5085	P	transmembrane transport	10	244	0.002<br>2	0.02
GO:000<br>6323	P	DNA packaging	5	64	0.002<br>2	0.02
GO:000<br>6779	P	porphyrin biosynthetic process	5	65	0.002<br>3	0.021
GO:001<br>5994	P	chlorophyll metabolic process	5	66	0.002<br>5	0.022
GO:004<br>3413	P	macromolecule glycosylation	5	66	0.002<br>5	0.022
GO:000<br>6486	P	protein amino acid glycosylation	5	66	0.002<br>5	0.022
GO:007	P	glycosylation	5	66	0.002	0.022

0085					5	
GO:0009101	P	glycoprotein biosynthetic process	5	66	0.0025	0.022
GO:0010557	P	positive regulation of macromolecule biosynthetic process	5	69	0.003	0.026
GO:0045935	P	positive regulation of nucleobase, nucleoside, nucleotide and nucleic acid metabolic process	5	69	0.003	0.026
GO:0051173	P	positive regulation of nitrogen compound metabolic process	5	70	0.0032	0.028
GO:0033014	P	tetrapyrrole biosynthetic process	5	72	0.0036	0.031
GO:0009416	P	response to light stimulus	17	596	0.0036	0.031
GO:0009100	P	glycoprotein metabolic process	5	73	0.0038	0.033
GO:0051603	P	proteolysis involved in cellular protein catabolic process	11	309	0.0038	0.033
GO:0070887	P	cellular response to chemical stimulus	14	452	0.004	0.034
GO:0006812	P	cation transport	12	357	0.004	0.034
GO:0044242	P	cellular lipid catabolic process	5	74	0.004	0.034
GO:0050832	P	defense response to fungus	6	108	0.0041	0.035
GO:004	P	cellular protein catabolic process	11	315	0.004	0.037

4257					4	
GO:0006470	P	protein amino acid dephosphorylation	5	76	0.0044	0.037
GO:0010604	P	positive regulation of macromolecule metabolic process	5	76	0.0044	0.037
GO:0010015	P	root morphogenesis	6	114	0.0053	0.044
GO:0007017	P	microtubule-based process	6	114	0.0053	0.044
GO:0033365	P	protein localization in organelle	5	80	0.0054	0.045
GO:0003824	F	catalytic activity	291	9638	8.80E-52	5.90E-49
GO:0016491	F	oxidoreductase activity	103	1463	2.50E-42	8.30E-40
GO:0046872	F	metal ion binding	109	1997	2.40E-35	5.50E-33
GO:0043167	F	ion binding	109	2087	9.90E-34	1.30E-31
GO:0043169	F	cation binding	109	2087	9.90E-34	1.30E-31
GO:0046906	F	tetrapyrrole binding	27	136	8.60E-22	9.60E-20
GO:0005488	F	binding	239	11258	1.70E-16	1.60E-14
GO:000	F	iron ion binding	22	161	5.70E	4.20E

5506					-15	-13
GO:0048037	F	cofactor binding	26	246	5.10E-15	4.20E-13
GO:0019842	F	vitamin binding	14	54	2.60E-13	1.70E-11
GO:0016168	F	chlorophyll binding	12	38	1.90E-12	1.10E-10
GO:0070279	F	vitamin B6 binding	12	41	4.00E-12	2.10E-10
GO:0030170	F	pyridoxal phosphate binding	12	41	4.00E-12	2.10E-10
GO:0000166	F	nucleotide binding	73	2267	8.10E-12	3.90E-10
GO:0017076	F	purine nucleotide binding	61	1713	1.10E-11	4.80E-10
GO:0020037	F	heme binding	15	97	2.50E-11	1.10E-09
GO:0032555	F	purine ribonucleotide binding	58	1631	3.90E-11	1.40E-09
GO:0032553	F	ribonucleotide binding	58	1631	3.90E-11	1.40E-09
GO:0046914	F	transition metal ion binding	55	1618	6.30E-10	2.20E-08
GO:0016984	F	ribulose-bisphosphate carboxylase activity	7	9	6.90E-10	2.30E-08
GO:001	F	carboxy-lyase activity	12	81	3.90E	1.20E

6831					-09	-07
GO:0051536	F	iron-sulfur cluster binding	9	37	8.10E-09	2.30E-07
GO:0051540	F	metal cluster binding	9	37	8.10E-09	2.30E-07
GO:0001883	F	purine nucleoside binding	49	1465	9.50E-09	2.50E-07
GO:0001882	F	nucleoside binding	49	1465	9.50E-09	2.50E-07
GO:0030554	F	adenyl nucleotide binding	49	1465	9.50E-09	2.50E-07
GO:0005198	F	structural molecule activity	30	659	1.60E-08	4.00E-07
GO:0035251	F	UDP-glucosyltransferase activity	12	94	1.80E-08	4.20E-07
GO:0016830	F	carbon-carbon lyase activity	14	141	2.20E-08	5.00E-07
GO:0032559	F	adenyl ribonucleotide binding	46	1384	3.30E-08	7.40E-07
GO:0005524	F	ATP binding	44	1377	1.90E-07	4.20E-06
GO:0016829	F	lyase activity	22	430	2.10E-07	4.40E-06
GO:0016853	F	isomerase activity	17	265	2.70E-07	5.40E-06
GO:000	F	monooxygenase activity	12	127	3.60E	7.10E

4497					-07	-06
GO:0005509	F	calcium ion binding	15	215	5.00E-07	9.60E-06
GO:0046527	F	glucosyltransferase activity	12	136	7.00E-07	1.30E-05
GO:0005507	F	copper ion binding	11	113	8.40E-07	1.50E-05
GO:0016705	F	oxidoreductase activity, acting on paired donors, with incorporation or reduction of molecular oxygen	14	198	1.00E-06	1.80E-05
GO:0016758	F	transferase activity, transferring hexosyl groups	18	340	1.70E-06	2.90E-05
GO:0003735	F	structural constituent of ribosome	22	494	1.90E-06	3.20E-05
GO:0016740	F	transferase activity	77	3321	2.10E-06	3.50E-05
GO:0016679	F	oxidoreductase activity, acting on diphenols and related substances as donors	7	42	3.60E-06	5.70E-05
GO:0050662	F	coenzyme binding	13	191	3.70E-06	5.80E-05
GO:0016682	F	oxidoreductase activity, acting on diphenols and related substances as donors, oxygen as acceptor	6	29	6.00E-06	9.10E-05
GO:0000287	F	magnesium ion binding	8	66	6.10E-06	9.10E-05
GO:0051213	F	dioxygenase activity	6	31	8.40E-06	0.00012
GO:001	F	antioxidant activity	11	150	1.10E	0.000

6209					-05	15
GO:000 8194	F	UDP-glycosyltransferase activity	13	219	1.50E -05	0.000 21
GO:001 6765	F	transferase activity, transferring alkyl or aryl (other than methyl) groups	11	158	1.70E -05	0.000 23
GO:003 1406	F	carboxylic acid binding	7	56	2.00E -05	0.000 26
GO:000 4364	F	glutathione transferase activity	7	57	2.20E -05	0.000 28
GO:008 0044	F	quercetin 7-O-glucosyltransferase activity	5	22	2.50E -05	0.000 32
GO:008 0043	F	quercetin 3-O-glucosyltransferase activity	5	26	5.00E -05	0.000 63
GO:001 6757	F	transferase activity, transferring glycosyl groups	20	544	7.50E -05	0.000 93
GO:001 9001	F	guanyl nucleotide binding	12	249	0.000 21	0.002 4
GO:003 2561	F	guanyl ribonucleotide binding	12	249	0.000 21	0.002 4
GO:000 5525	F	GTP binding	12	249	0.000 21	0.002 4
GO:001 6684	F	oxidoreductase activity, acting on peroxide as acceptor	8	127	0.000 45	0.005 1
GO:000 4601	F	peroxidase activity	8	127	0.000 45	0.005 1
GO:001	F	protein disulfide oxidoreductase activity	5	46	0.000	0.006

5035					56	2
GO:0003924	F	GTPase activity	6	72	0.00059	0.0065
GO:0016667	F	oxidoreductase activity, acting on sulfur group of donors	7	109	0.0009	0.0098
GO:0016709	F	oxidoreductase activity, acting on paired donors, with incorporation or reduction of molecular oxygen, NADH or NADPH as one donor, and incorporation of one atom of oxygen	5	56	0.0013	0.014
GO:0016787	F	hydrolase activity	67	3468	0.0017	0.018
GO:0015036	F	disulfide oxidoreductase activity	5	60	0.0017	0.018
GO:0004252	F	serine-type endopeptidase activity	5	66	0.0025	0.025
GO:0042625	F	ATPase activity, coupled to transmembrane movement of ions	5	68	0.0028	0.028
GO:0017171	F	serine hydrolase activity	8	186	0.0044	0.042
GO:0008483	F	transaminase activity	5	76	0.0044	0.042
GO:0008236	F	serine-type peptidase activity	8	186	0.0044	0.042
GO:0016614	F	oxidoreductase activity, acting on CH-OH group of donors	8	188	0.0047	0.044
GO:0016769	F	transferase activity, transferring nitrogenous groups	5	77	0.0047	0.044
GO:001	C	integral to membrane	99	395	6.40E	3.10E

6021					-87	-84
GO:0044425	C	membrane part	129	1360	1.10E-67	2.70E-65
GO:0031224	C	intrinsic to membrane	100	806	1.20E-61	1.90E-59
GO:0016020	C	membrane	158	4068	3.60E-35	4.40E-33
GO:0009579	C	thylakoid	44	376	3.20E-26	3.20E-24
GO:0044422	C	organelle part	104	2562	9.80E-24	8.00E-22
GO:0044446	C	intracellular organelle part	103	2561	3.30E-23	2.30E-21
GO:0055044	C	symplast	17	19	5.80E-23	3.20E-21
GO:0009506	C	plasmodesma	17	19	5.80E-23	3.20E-21
GO:0005911	C	cell-cell junction	17	21	1.90E-22	9.30E-21
GO:0034357	C	photosynthetic membrane	35	273	3.00E-22	1.30E-20
GO:0009521	C	photosystem	22	66	3.80E-22	1.60E-20
GO:0030054	C	cell junction	17	23	5.70E-22	2.10E-20
GO:000	C	cytoplasm	176	6822	4.00E	1.40E

5737					-19	-17
GO:0005622	C	intracellular	221	9671	1.30E-18	4.40E-17
GO:0044424	C	intracellular part	213	9302	8.00E-18	2.40E-16
GO:0005623	C	cell	294	15217	5.70E-16	1.60E-14
GO:0009523	C	photosystem II	15	45	1.60E-15	4.40E-14
GO:0031090	C	organelle membrane	46	842	4.40E-15	1.10E-13
GO:0032991	C	macromolecular complex	78	2180	7.30E-15	1.80E-13
GO:0044464	C	cell part	290	15217	9.60E-15	2.20E-13
GO:0009522	C	photosystem I	12	22	1.00E-14	2.30E-13
GO:0044436	C	thylakoid part	28	307	1.40E-14	2.90E-13
GO:0044459	C	plasma membrane part	23	222	2.90E-13	5.80E-12
GO:0009534	C	chloroplast thylakoid	25	290	1.20E-12	2.30E-11
GO:0031976	C	plastid thylakoid	25	293	1.40E-12	2.70E-11
GO:003	C	organelle subcompartment	25	295	1.60E	3.00E

1984					-12	-11
GO:0005576	C	extracellular region	24	285	5.20E-12	9.10E-11
GO:0044444	C	cytoplasmic part	146	6289	7.30E-12	1.20E-10
GO:0005829	C	cytosol	42	912	1.40E-11	2.30E-10
GO:0044434	C	chloroplast part	37	746	3.30E-11	5.30E-10
GO:0042651	C	thylakoid membrane	21	244	7.70E-11	1.20E-09
GO:0055035	C	plastid thylakoid membrane	20	231	2.00E-10	2.80E-09
GO:0009535	C	chloroplast thylakoid membrane	20	231	2.00E-10	2.80E-09
GO:0044435	C	plastid part	37	867	1.80E-09	2.50E-08
GO:0009941	C	chloroplast envelope	20	265	1.80E-09	2.50E-08
GO:0043229	C	intracellular organelle	167	8149	2.10E-09	2.80E-08
GO:0043226	C	organelle	167	8155	2.30E-09	2.90E-08
GO:0043234	C	protein complex	49	1443	6.00E-09	7.50E-08
GO:000	C	plastid envelope	20	331	6.00E	7.40E

9526					-08	-07
GO:004 3232	C	intracellular non-membrane-bounded organelle	37	1040	1.60E -07	1.80E -06
GO:004 3228	C	non-membrane-bounded organelle	37	1040	1.60E -07	1.80E -06
GO:000 5774	C	vacuolar membrane	11	100	2.70E -07	3.10E -06
GO:003 1975	C	envelope	26	595	3.10E -07	3.40E -06
GO:003 1967	C	organelle envelope	26	595	3.10E -07	3.40E -06
GO:004 4437	C	vacuolar part	11	104	3.90E -07	4.20E -06
GO:000 5840	C	ribosome	24	524	4.00E -07	4.20E -06
GO:003 0529	C	ribonucleoprotein complex	28	703	6.50E -07	6.60E -06
GO:000 9654	C	oxygen evolving complex	6	21	1.20E -06	1.20E -05
GO:002 2626	C	cytosolic ribosome	18	336	1.40E -06	1.40E -05
GO:004 8046	C	apoplast	13	182	2.20E -06	2.10E -05
GO:000 9570	C	chloroplast stroma	15	249	2.80E -06	2.60E -05
GO:004	C	cytosolic part	18	360	3.60E	3.30E

4445					-06	-05
GO:0022625	C	cytosolic large ribosomal subunit	11	162	2.10E-05	0.00019
GO:0009507	C	chloroplast	63	2740	2.60E-05	0.00023
GO:0033279	C	ribosomal subunit	17	389	3.40E-05	0.0003
GO:0009532	C	plastid stroma	15	322	5.00E-05	0.00043
GO:0005773	C	vacuole	16	383	9.60E-05	0.00081
GO:0009536	C	plastid	63	2965	0.00023	0.0019
GO:0005874	C	microtubule	5	38	0.00025	0.002
GO:0044431	C	Golgi apparatus part	8	118	0.00028	0.0022
GO:0015934	C	large ribosomal subunit	11	225	0.00033	0.0026
GO:0005886	C	plasma membrane	36	1456	0.00042	0.0033
GO:0043227	C	membrane-bounded organelle	133	7622	0.00055	0.0042
GO:0043231	C	intracellular membrane-bounded organelle	132	7615	0.00075	0.0057
GO:000	C	endoplasmic reticulum membrane	5	64	0.002	0.016

5789					2	
GO:0005794	C	Golgi apparatus	11	293	0.0026	0.019
GO:0042175	C	nuclear envelope-endoplasmic reticulum network	5	67	0.0027	0.019
GO:0000785	C	chromatin	5	71	0.0034	0.024
GO:0005618	C	cell wall	13	403	0.0039	0.027
GO:0031226	C	intrinsic to plasma membrane	5	74	0.004	0.027
GO:0030312	C	external encapsulating structure	13	407	0.0042	0.028
GO:0044432	C	endoplasmic reticulum part	5	75	0.0042	0.028
GO:0005783	C	endoplasmic reticulum	14	466	0.0051	0.034
GO:0010287	C	plastoglobule	5	81	0.0057	0.037

Note: Enriched GO terms were generated in agriGO with customized annotation as reference. p-values were converted with Z-score generated by z-test. FDR were generated as p-value with multiple test correction.

Supplementary Table s6. Comparison of protein and mRNA expression pattern of 15 candidate proteins (genes) at different developmental stages

		**iTRAQ（8 × plex）**								**RT-PCR**							
		**Danfeng-2**				**Cabernet Sauvignon**				**Danfeng-2**				**Cabernet Sauvignon**			
**Protein accession**	**Description**	**Green hard**	**Before Veraison**	**Veraison**	**Ripe**	**Green hard**	**Before Veraison**	**Veraison**	**Ripe**	**Green hard**	**Before Veraison**	**Veraison**	**Ripe**	**Green hard**	**Before Veraison**	**Veraison**	**Ripe**
A5BGJ0	DFR	1.000	0.827	0.751	1.115	2.186	0.726	1.878	1.519	0.095	0.070	0.075	0.148	0.139	0.037	1.000	0.137
A5BMU2	CHI1	1.000	0.956	0.995	0.903	1.898	0.943	1.536	1.756	0.106	0.140	0.080	0.140	0.192	0.046	1.000	0.409
A5ANT9	CHI2	1.000	0.900	0.899	0.752	1.561	0.910	1.116	1.642	0.135	0.091	0.141	0.094	0.184	0.035	1.000	0.289
A2ICC9	LDOX	1.000	0.971	1.040	0.993	1.977	0.946	1.973	1.758	0.114	0.080	0.114	0.158	0.210	0.104	1.000	0.242
G4XGW2	CHS	1.000	0.825	0.810	0.706	1.827	0.733	3.971	2.818	0.020	0.007	0.006	0.003	0.113	0.001	1.000	0.248
Q2L7J4	F35'H1	1.000	1.125	0.756	1.012	1.759	1.057	0.993	1.972	0.020	0.004	0.020	0.002	0.068	0.003	1.000	0.086
F6HA89	F35'H2	1.000	0.928	1.120	1.298	1.909	1.324	1.300	2.962	1.029	0.915	0.494	0.651	0.565	0.534	0.333	1.128
A5BZR3	F3H	1.000	0.945	1.162	0.921	1.956	0.918	1.622	1.704	0.029	0.028	0.055	0.022	0.178	0.007	1.000	0.132
A5BRL4	C4H	1.000	0.954	1.046	0.904	1.230	0.939	1.360	1.786	0.047	0.056	0.325	0.138	0.144	0.061	1.000	0.428
A5BIH9	RSGT	1.000	0.815	1.113	1.994	0.997	0.875	0.889	0.938	0.402	0.416	0.581	2.564	1.286	0.211	0.148	0.037
A5BBF4	UBC32	1.000	0.947	1.029	1.334	0.985	1.011	1.662	1.320	0.764	0.580	0.488	0.954	0.278	0.361	0.590	1.000
A5BFQ1	LRR	1.000	0.987	0.973	0.835	1.013	0.781	1.997	1.609	0.528	0.502	0.798	0.272	1.173	0.649	1.893	0.245
A5AI47	GRP	1.000	0.760	1.667	7.442	1.657	1.177	0.965	4.355	0.171	0.047	1.738	0.250	0.000	0.007	0.068	1.000
A3FA68	PIP2;3	1.000	0.906	0.897	1.833	1.000	1.032	2.299	2.423	0.063	0.005	0.007	0.341	0.116	0.097	0.792	1.000
D7TA35	USP	1.000	1.117	1.003	1.437	1.059	1.124	1.160	0.903	1.368	1.553	1.125	1.013	1.286	0.657	1.587	0.602

Note: RT-PCR data are log2 normalized 2−ΔΔCt values. Relationships of proteins with qRT-PCR transcripts are indicated.
